# Unraveling Terminal Cobalt–Antimony Double Bond: Breaking New Ground in Heavy Pnictogen Multiple Bonds

**DOI:** 10.1002/anie.8012161

**Published:** 2026-07-23

**Authors:** Daniel Meleschko, Prasenjit Palui, Rosa M. Gomilia, Morgan J. McKee, Gregor Schnakenburg, Nikolay Kornienko, Antonio Frontera, Alessandro Bismuto

**Affiliations:** ^1^ Institute of Inorganic Chemistry University of Bonn Bonn Germany; ^2^ Departament de Química Universitat De Les Illes Balears, Palma Baleares Spain

**Keywords:** cycloaddition, distibenes, heavy double bonds, stibinidene‐transfer reagent

## Abstract

Terminal pnictinidene complexes featuring heavy group 15 elements (Sb, Bi) doubly bonded to transition metals remain exceptionally elusive compared to their lighter congeners. Herein, we demonstrate that N‐heterocyclic carbenes (NHCs) can effectively cleave the Sb═Sb double bond of distibenes to yield monomeric, zwitterionic stibinide species. Subsequent transfer of the antimony moiety to a cobalt center generates a terminal cobalt–stibinidene complex. Structural analysis reveals a very short Co─Sb bond distance, which is corroborated by Wiberg and Mayer bond indices as well as frontier molecular orbital and natural bond orbital analyses, providing compelling evidence for an unprecedented covalent Co═Sb double bond. In addition, we demonstrate that this compound reacts with an alkyne in a (2π+2π) cycloaddition and with phenyl azide to form a three‐membered ring, providing initial insight into its reactivity and drawing parallels with reactivity patterns established for lighter phosphinidene analogs. This work establishes a rational route to stabilize heavy‐element multiple bonds *via* a controlled and operationally‐simple distibene cleavage.

## Graphical abstract

N‐heterocyclic carbenes (NHCs) function as “molecular scissors,” cleaving otherwise inert Sb═Sb bonds in distibenes to generate monomeric, zwitterionic stibinide species. These intermediates act as efficient stibinidene transfer reagents, granting access to a rare terminal cobalt–stibinidene complex. Structural and computational analysis indicate a covalent Co═Sb double bond with pronounced olefin‐like π character, as further evidenced by its preliminary reactivity profile.

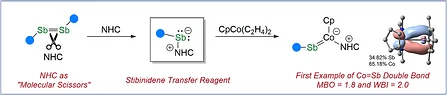

## 1 Introduction

**FIGURE 1:**
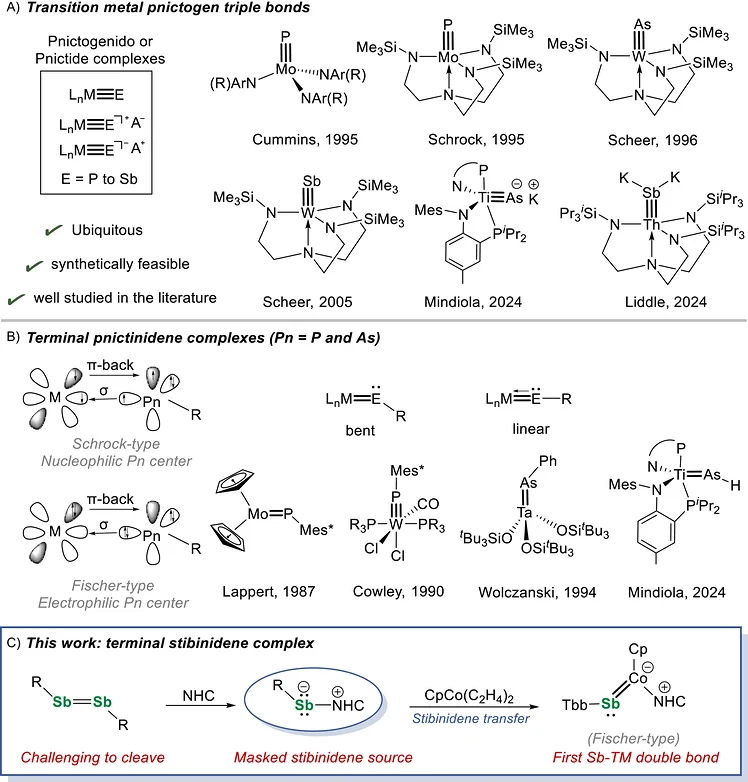
(A) Selected examples of pnictogenido complexes reported in the literature; (B) Selected examples of pnictinidene complexes reported in the literature; (C) Our approach to cleave heavy pnictogen double bonds to synthesize a terminal stibinidene complex; TM = transition metal; R = any alkyl or aryl group; Mes* = 2,4,6‐tri‐tert‐butyl phenyl; Mes = 2,4,6‐trimethyl phenyl; Tbb = 2,6‐[CH(SiMe_3_)_2_]_2_‐4‐(^*t*^Bu)‐C_6_H_2_; NHC = N‐heterocyclic carbene. Assignment of complex **4** to a Fischer‐type category is based on natural population analysis (NPA).

The discovery of olefin metathesis and the subsequent development of carbon–transition‐metal multiple‐bond chemistry have profoundly transformed the landscape of modern organic and organometallic synthesis [1]. This success has, in turn, stimulated efforts to incorporate heavier main‐group elements into transition‐metal frameworks. In particular, the chemistry of group 14 elements (Si, Ge, Sn) has matured considerably, giving rise to a diverse array of ylidene (MG = TM) and ylidyne (MG≡TM) complexes whose reactivity expands upon that of classical metal–carbon multiply bonded systems [2, 3, 4, 5, 6, 7, 8, 9]. In contrast, in group 15, a fundamental imbalance in multiple bonds persists. Transition‐metal pnictogen triple bonds, featuring a monocoordinated pnictogen, have been known even longer than metal–carbyne complexes [10, 11]. Among them, nitrido complexes, reported in the early 1970s, have been used in nitrogen‐atom‐transfer chemistry and even applicable in ammonia synthesis through sequential protonation and electron‐transfer steps [12, 13]. This chemistry extends beyond nitrogen to heavier congeners with pioneering examples of phosphido, arsido, and stibido complexes across early and late transition metals and even actinide (Figure 1A) [10, 14, 15, 16, 17, 18]. However, their double‐bonded counterparts remain strikingly rare for the heavier members of the group [12, 13]. This disparity is especially evident when comparing phosphorus to its heavier congeners. Terminal phosphinidene complexes (TM = P) have been extensively studied and displayed a vast reactivity profile that includes (2π+2π) cycloadditions with alkynes, phosphaalkene formation, and moiety transfer [19, 20]. Early milestones include the pioneering work of Lappert and co‐workers, who isolated the first bent, doubly bonded Mo– and W–phosphinidene complexes, followed by Cowley's synthesis of the first linear phosphinidene using Mes*PCO as a precursor [21, 22]. Since then, the chemistry of phosphinidenes has flourished, revealing rich reactivity such as cycloadditions with alkynes, phosphaalkene formation, and transfer of free phosphinidene (Figure 1B) [23].

Yet, this versatility diminishes drastically down the chemical group; while few arsinidene complexes are known [24, 25, 26], terminal stibinidene (TM = Sb) and bismuthinidene (TM = Bi) complexes remain essentially unknown. A major barrier lies in identifying suitable precursors capable of delivering a free Pn–R fragment [27, 28, 29]. To address this gap, we envisaged the use of distibenes, known for more than 25 years, as a potential source for generating unprecedented transition‐metal–antimony double bonds [30]. We anticipated that NHC could promote a controlled cleavage of the distibene double bond, in a similar approach to that of Schulz and co‐workers [31], thereby generating a perfect stibinidene synthon and enabling transfer to a suitable transition‐metal precursor through cleavage of the Sb─C^NHC^ bond. Herein, we report a rational, stepwise synthetic route to a rare covalent Co═Sb double bond with an olefin‐like π‐symmetry which exhibits the hallmark [2π+2π] cycloaddition reactivity with alkynes. More broadly, this work establishes a general and operationally‐simple blueprint for accessing heavy‐element double bonds, effectively closing a long‐standing gap in the periodic table's multiple‐bonding landscape (Figure 1C).

## 2 Results and Discussion

**FIGURE 2:**
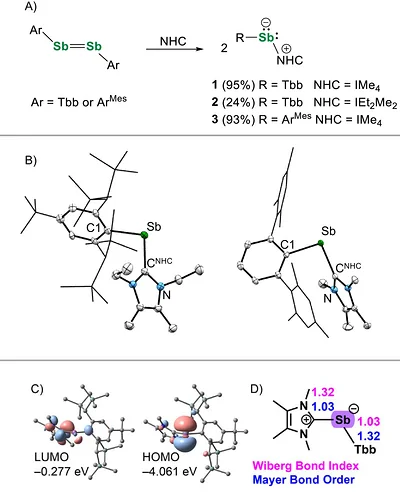
(A) General reaction scheme for the formation of stibinide complexes; (B) Crystal structures of complex **2** and **3**. H atoms omitted for clarity; (C) FMOs of complex **1**; and (D) Wiberg bond index (pink) and Mayer bond order (blue) values for both Sb–C bonds at the x2c‐PW6B95‐D4/x2c‐QZVPPall//TPSS‐D4/def2‐TZVP level of theory.

Our investigation started with the evaluation of NHC as reagents for controlled cleavage of Sb═Sb bond in the sterically protected Sb_2_Tbb_2_ (Tbb = 2,6‐[CH(SiMe_3_)_2_]_2_‐4‐(*^t^* Bu)‐C_6_H_2_). The addition of IMe_4_ (IMe_4_ = 1,3,4,5‐tetramethylimidazol‐2‑ylidene) to a yellow‐orange suspension of distibene led to an immediate color change to deep red (Figure 2A). The ^1^H NMR spectrum of the reaction mixture after ten minutes confirmed an almost quantitative conversion into complex **1**, which was isolated in 95% yield as an orange‐red crystalline solid and fully characterized by NMR and UV/vis spectroscopy. Single‐crystal X‐ray analysis confirmed the NHC‐supported stibinide structure motif of compound **1**. However, the high crystallographic symmetry limited the precision of the structural parameters, precluding a detailed discussion of bonding metrics.

To overcome this problem, we decided to employ a different NHC ligand with the aim of lowering the symmetry group. In contrast to IMe_4_, the reaction of distibene with IEt_2_Me_2_ at room temperature proceeded sluggishly and required higher temperature (85°C). After 3 h of heating a consumption of 50% of starting material was observed, which unfortunately could not be improved even using different solvents or temperatures (see SI for further details). Compound **2** was isolated as an orange‐red crystalline solid in 24% yield and characterized by single‐crystal x‐ray diffraction analysis. As anticipated, this time the compound crystallized in a lower symmetry group allowing us to discuss all the bonding parameters. The Sb─C^NHC^ (2.192(4) Å) bond distance and the C1–Sb–C^NHC^ (99.58(16)°) angle are in good agreement with those reported for NHC‐supported stibinidenes by Schulz and co‐workers and consistent with a formal Sb─C single bond [31, 32]. However, they differ significantly from those reported for CAAC/DAC‐stabilized stibinidenes in the literature, as these carbenes are considerably more π accepting than classical NHCs and imparts partial Sb═C double bond character, thereby provide enhanced stabilization to the stibinidenes [33, 34]. Encouraged by the pronounced ligand effect, we next examined whether an analogous transformation could be achieved using a different type of distibene, Sb_2_Ar^Mes^_2_ (Ar^Mes^ = 2,6‐(2,4,6‐Me_3_‐C_6_H_2_)_2_‐C_6_H_3_). Upon addition of IMe_4_ to a solution of distibene, we noted an immediate color change from yellow to deep red. Compound **3** was isolated as an orange‐red crystalline solid in 93% yield (Figure 2A,B). Single‐crystal x‐ray analysis confirmed the formation of the stibinidene motif, which is isostructural with compounds **1** and **2**. Attempts to extend the reaction to bulkier NHCs, *e.g*. IPr_2_Me_2_, resulted in no observable product formation, highlighting the critical role of steric effects in this transformation [35].

To gain deeper insight into the electronic structure of these species, we performed DFT calculations on the three new complexes. Given the high similarity between them, we discuss only the results for **1** here, while the data for **2** and **3** are provided in Figure S7. Analysis of the frontier molecular orbitals (Figure 2C) reveals that the HOMO corresponds to the lone pair on the antimony atom, while the LUMO is localized on the NHC framework. This electronic topography aligns with the expected nucleophilicity of the stibinide motif. The bonding situation is further elucidated by the Wiberg bond indices (WBI) and Mayer bond orders (MBO) presented in Figure 2D. The values for the Sb–C interactions are close to unity (approx. 1.0 and 1.3, respectively), which effectively rules out the formation of a C═Sb double bond. Instead, these metrics support the zwitterionic resonance structure depicted in Figure 2D (NHC^+^–Sb^–^). Furthermore, NBO analysis identified a specific σ‐bonding orbital localized between the Sb center and the carbene carbon (see Figure S8). This confirms that the NHC effectively “unlocks” the distibene by generating a monomeric, nucleophilic compound that could serve as a stibinidene synthon for transition‐metal transfer.

**FIGURE 3:**
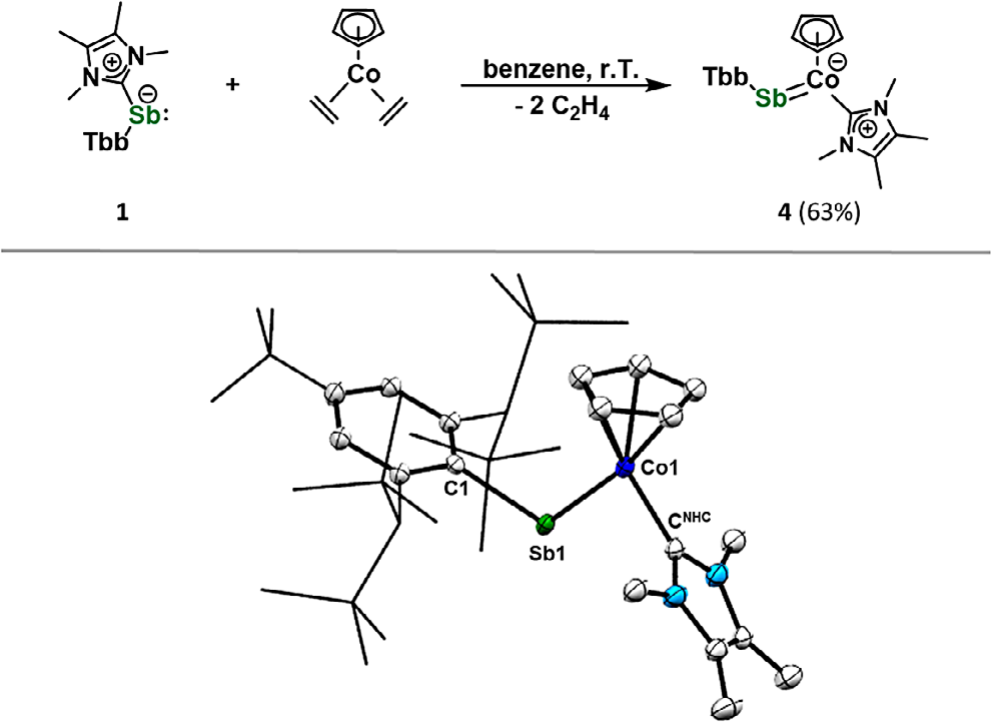
Reaction scheme for the reaction of stibinide **1** with CpCo(C_2_H_4_)_2_ and the corresponding crystal structure. Ellipsoids are drawn at 30% probability and hydrogen atoms are omitted for clarity.

Since phosphinidene complexes have been key synthons for compounds featuring a multiple bond, we focused on the reaction of compound **1** with transition‐metal complexes using Jonas’ complex CpCo(C_2_H_4_)_2_. The reaction proceeded smoothly at room temperature, accompanied by the displacement of ethylene and a distinct color change to brown, yielding complex **4** as a brown crystalline solid in 63% yield (Figure 3). Single‐crystal x‐ray diffraction analysis confirmed the formation of a two‐legged piano‐stool complex featuring a very short Sb–Co bond of 2.4070(12) Å. This bond length is significantly shorter than those reported for typical Sb–Co single bonds (∼2.50 Å) and is closer to the value expected for an Sb═Co double bond (Σr(Sb═Co)_cov._ = 2.36 Å) [36, 37, 38]. Furthermore, the V‐shaped antimony center with an angle of C^Tbb^−Sb−Co = 112.37(18) ° is well comparable to that observed for typical terminal phosphinidene complexes found in the CSD database (∼115 °), supporting multiple‐bond character in the Sb−Co interaction [39, 40]. The Co−C^NHC^ bond length of 1.920(8) Å is consistent with those reported for Co(I)−NHC complexes in the literature [41, 42].

**FIGURE 4:**
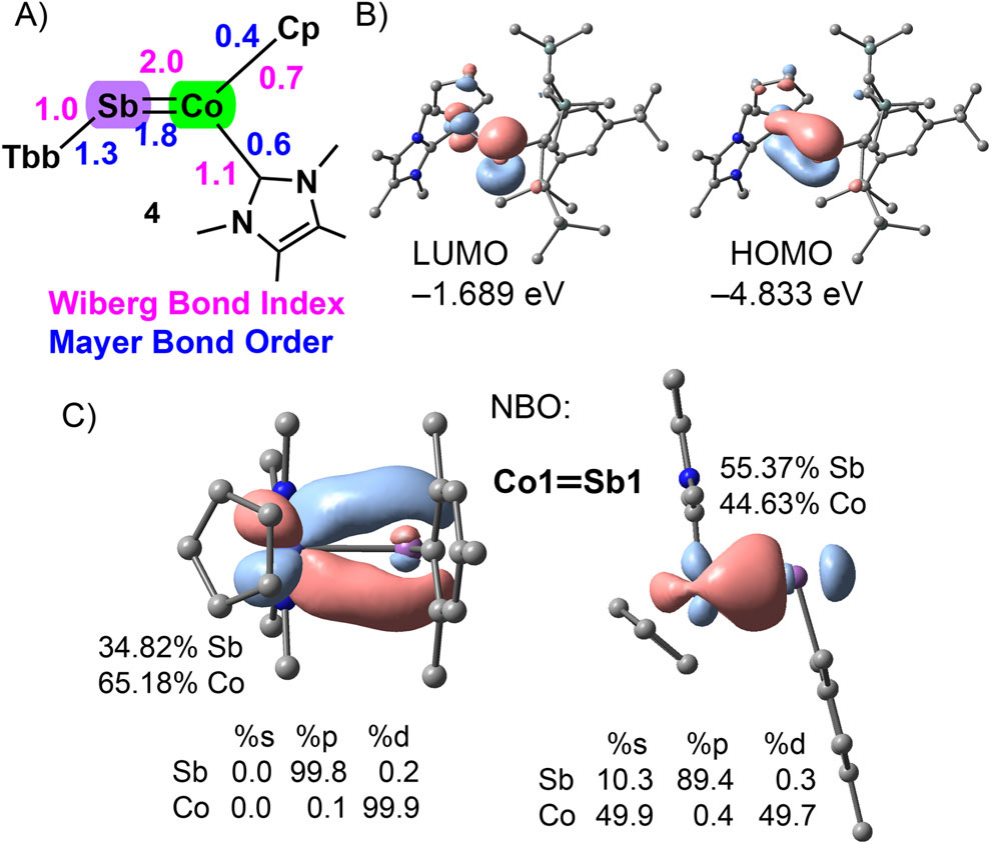
Electronic structure of complex **4** calculated at the x2c‐PW6B95‐D4/x2c‐QZVPPall//TPSS‐D4/def2‐TZVP level. (A) Wiberg (pink) and Mayer (blue) bond indices support a double bond; (B) Frontier molecular orbitals (HOMO and LUMO) showing the π‐bonding and π*‐antibonding character; (C) Natural bond orbitals (NBOs) depicting the π‐ (left) and σ‐ (right) components of the Co═Sb bond with their respective atomic compositions.

To elucidate the nature of the cobalt antimony interaction, we performed DFT calculations on complex **4** at the x2c‐PW6B95‐D4/x2c‐QZVPPall//TPSS‐D4/def2‐TZVP level of theory. The optimized structure reproduces the experimental geometry well, with a calculated Co–Sb bond length of 2.38 Å (see Figure S9). The slight shortening compared to the experimental value (2.40 Å) is attributed to thermal disorder observed in the x‐ray structure. The calculations provide strong evidence for the double‐bond character of the cobalt–antimony interaction (Figure 4A). The WBI and MBO are found to be 2.0 and 1.8, respectively, values that are consistent with a formal Co═Sb double bond. This bonding situation is further corroborated by the frontier molecular orbitals (FMOs) depicted in Figure 4B. The HOMO and LUMO correspond to the bonding π and antibonding π* orbitals, respectively, and are strictly localized on the Co═Sb bond. This electronic topology is reminiscent of the π‐system found in olefins, reinforcing the assignment of a double bond. To gain deeper insight into the atomic contributions, a NBO analysis was performed (Figure 4C). This reveals two distinct interactions: a σ‐bond and a π‐bond. The σ‐bond (Figure 4C, right) is polarized toward antimony (approx. 55% Sb, 45% Co) and exhibits significant *p*‐character on Sb. In contrast, the π‐bond (Figure 4C, left) is polarized toward cobalt (approx. 65% Co, 35% Sb) and is formed primarily by the overlap of a d‐orbital on Co with a p‐orbital on Sb. The multiple bond character and the strong covalency have also been confirmed using natural orbitals for chemical valence (ETS‐NOCV) with two main deformation density pairs along with a total orbital stabilization energy of −110.03 kcal/mol (see Table S1). Ultimately, the specific localization of the frontier orbitals (Figure 4B) on the heavy‐atom core anticipates that the reactivity of complex **4** will be centered at the Co═Sb functionality.

**FIGURE 5:**
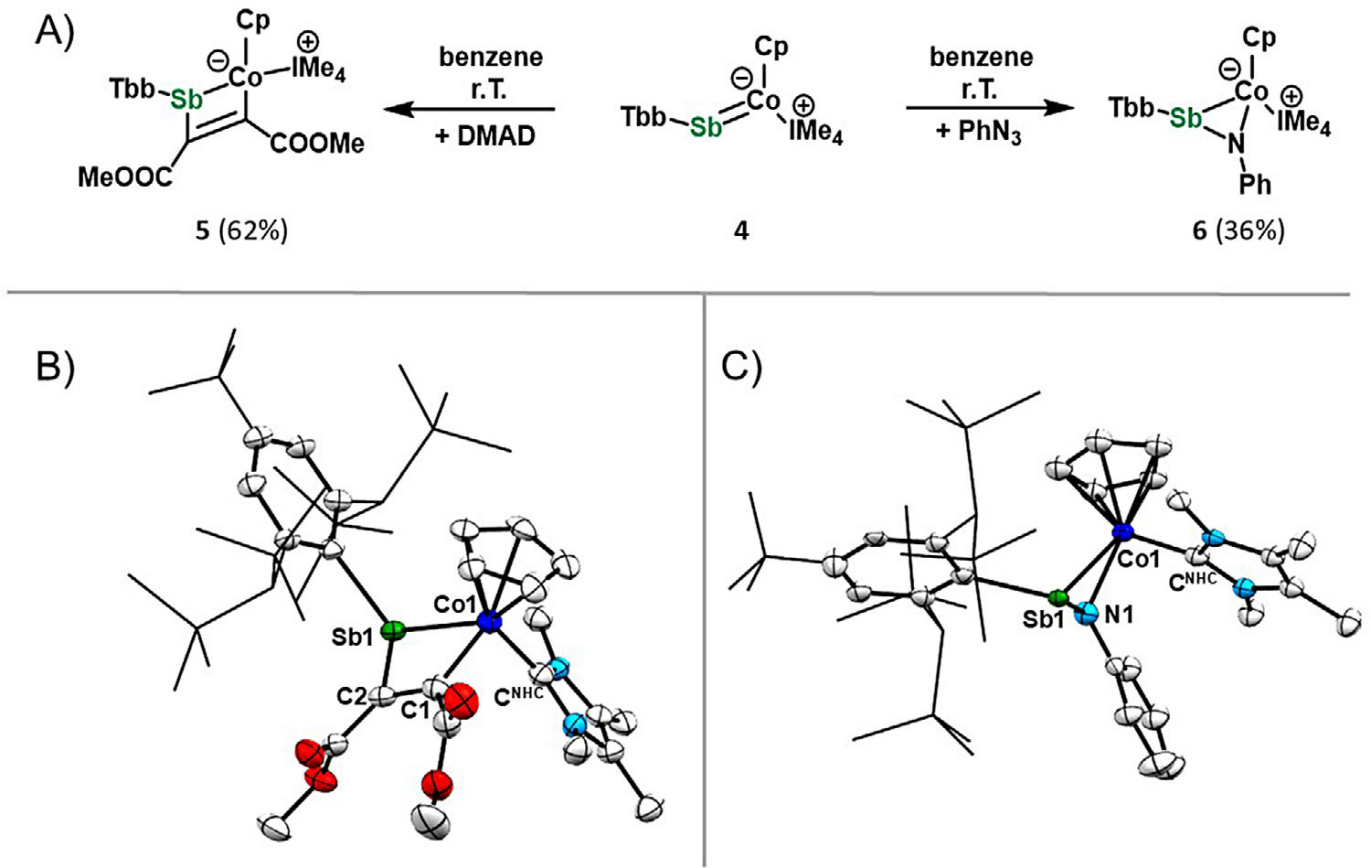
(A) Reaction scheme for the reaction to form complex **5** and **6**; (B) and (C) Single‐crystal x‐ray structures of complex **5** and **6**. Ellipsoids are drawn at 30% probability and hydrogen atoms are omitted for clarity.

Based on this information and to further support the double bond character, we evaluated the reactivity of this new reagent in a (2π+2π) cycloaddition reaction with an electron deficient alkyne, namely dimethyl acetylenedicarboxylate (DMAD) (Figure 5A). Upon mixing of the two reagents in benzene a selective conversion to a new product was observed by NMR. The new compound was isolated as grey‐red crystalline solid in 62% yield and 1D and 2D NMR characterization hinted to a successful cyclization (see Supporting Information for further details). The identity of the compound was then further supported by single‐crystal x‐ray diffraction, which unambiguously confirmed the formation of a 4‐membered ring **5** (Figure 5B) [43]. In solid‐state, it features a planar four‐membered core (∠Co–C1–Sb–C2 = 175.9(4)°), with a pyramidal antimony center (Σ∠Sb = 289.2°) and two planar carbon centers (Σ∠C = 360°). The Sb–Co bond length (2.6015(7) Å) is markedly elongated compared to that of its precursor and falls within the range expected for a typical Sb–Co single bonds observed in Co–Sb‐containing heterocycles [44, 45]. To further evaluate the reactivity scope and synthon potential of complex **4**, we investigated its interaction with phenyl azide. This process resulted in the formation of a novel three‐membered metallacycle **6** in 36% yield, featuring a constrained Sb–Co–N core, which was unequivocally characterized by single‐crystal x‐ray diffraction analysis (Figure 5C). Compound **6** features a three‐membered heterocyclic core comprising of two pyramidal pnictogen centers (Σ∠N = 327° and Σ∠Sb = 266°), bonded to cobalt with bond distances of 2.549 (1) Å (Sb–Co) and 2.005 (1) Å (N–Co). The Sb–Co distance is considerably elongated relative to that in its precursor but remains consistent with Co–Sb single bonds reported for related cobalt complexes featuring Co–Sb_2_ core [44, 45]. Likewise, the Sb–N bond length (2.110(1) Å) falls within the expected range for an Sb–N single bond and well comparable to those previously reported for azadistibiranes in the literature [46, 47]. The N–Co distance is slightly elongated relative to those observed in related cobalt–diazene complexes (∼1.90 Å) across different cobalt oxidation states [48, 49]. This slight elongation might originate due to ring strain induced by the three‐membered heterocycle containing the heavier antimony atom. The limited stability of compound **6** led to the formation of 1,3,2,4‐dioxadistibetane derivative (complex **7**) as a side product during NMR characterization (see Supporting Information for further details).

## 3 Conclusion

In conclusion, we have established a novel and efficient synthetic protocol for accessing terminal stibinidene complexes, a class of compounds that has long remained elusive. By leveraging the nucleophilic cleavage of distibenes with N‐heterocyclic carbenes, we generated discrete zwitterionic stibinides that serve as competent transfer reagents. This strategy enabled the isolation of complex **4**, featuring a rare terminal cobalt–antimony double bond. Computational analysis unequivocally supports the presence of a Co═Sb double bond with significant covalent character and olefin‐like electronic topology. The synthetic utility of this species was demonstrated through its versatile reactivity profile; beyond the (2π+2π) cycloaddition with DMAD, the potential of complex **4** was further validated by its reaction with phenyl azide, which resulted in the selective formation of a three‐membered ring. These results highlight the ability of complex **4** to serve as a synthon for accessing complex heavy‐pnictogen architectures. Future studies will explore the extension of our molecular scissors approach to dibismuthenes. Although navigating the pronounced relativistic effects of bismuth presents some challenges, this trajectory remains a rational pathway toward completing the group 15 homologous series and effectively closing a long‐standing gap in the multiple‐bonding landscape.

## Author Contributions

**Daniel Meleschko**: writing – review and editing, conceptualization, methodology, investigation. **Prasenjit Palui**: conceptualization, writing – review and editing, methodology, investigation. **Rosa M. Gomilia**: data curation, writing – review and editing. **Morgan J. Mckee**: data curation, writing – review and editing. **Gregor Schnakenburg**: writing – review and editing, data curation. **Nikolay Kornienko**: writing – review and editing. **Antonio Frontera**: writing – review and editing, data curation, formal analysis. **Alessandro Bismuto**: conceptualization, funding acquisition, writing – original draft, writing – review and editing, project administration, resources, supervision, validation, visualization.

## Conflicts of Interest

The author declares no conflicts of interest.

## Supporting information




**Supporting File**: The authors have cited additional references within the Supporting Information [50, 51, 52, 53, 54, 55, 56, 57, 58, 59, 60, 61, 62, 63, 64, 65, 66, 67, 68, 69, 70, 71, 72, 73].

## Data Availability

The data that supports the findings of this study are available in the Supporting Information of this article.
