# Supplementary material for: Unraveling Terminal Cobalt–Antimony Double Bond: Breaking New Ground in Heavy Pnictogen Multiple Bonds
Source: Angew Chem Int Ed Engl. 2026 Jul 23;65(37):e8012161. doi: 10.1002/anie.8012161 (PMC13549010; doi:10.1002/anie.8012161)
Supplement: Supplementary file 1 [file ANIE-65-e8012161-s001.pdf]

Unravelling Terminal Cobalt–Antimony Double Bond: Breaking New Ground in Heavy Pnictogen Multiple Bonds

Daniel Meleschko, Prasenjit Palui, Rosa M. Gomila, Morgan J. McKee, Gregor Schnakenburg, Nikolay Kornienko, Antonio Frontera and Alessandro Bismuto*

*bismuto@uni-bonn.de

Table of Contents

1. General information ... 3
2. Syntheses of Stibinidines and Sb=Co complex ... 4
   2.1. Synthesis of complex **1** ... 4
   2.2. Synthesis of complex **2** ... 5
   2.3. Synthesis of complex **3** ... 6
   2.4. Synthesis of complex **4** ... 7
   2.5. Synthesis of complex **5** ... 8
   2.6. Synthesis of complex **6** ... 9
3. UV/Vis Spectroscopic Studies ... 10
4. Electrochemical Studies ... 12
   4.1. Scan Rate Dependence of Complex **4** ... 14
5. Computational methods and DFT calculations ... 16
   5.1. Computational methods ... 16
   5.2. DFT calculations ... 16
6. Single-crystal X-ray diffraction analysis ... 25
7. NMR spectra ... 30
8. References ... 36

1. General information

All experiments were carried out in a glovebox (UNILAB, MBraun) under an argon atmosphere or using standard Schlenk techniques. Benzene, tetrahydrofuran, toluene, diethylether, *n*-pentane and *n*-hexane were dried over sodium and were distilled and stored over molecular sieves (3 Å) prior to use. Acetonitrile was distilled upon stirring over CaH_2 and stored over molecular sieves (3 Å) prior to use.

Unless otherwise specified, all reagents were used as received from commercial suppliers (ABCR, AcrosOrganics, Alfa Aesar, BLDpharm, Sigma Aldrich, Thermo Fisher Scientific). All pnictogen halides PnX_3 were purified by sublimation under reduced pressure and stored under Ar atmosphere prior to use. Sb_2Tbb_2,[1] $Sb_2Ar^{Mes}{}_2$,[2] IMe_4,[3] IEt_2Me_2[3] and $CpCo(C_2H_4)_2$[4] were synthesized according to literature.

^1H and $^{13}C\{^1H\}$ NMR spectra were recorded in C_6D_6 at 25 °C using a Bruker Avance I 300 MHz, Bruker Avance I 400 MHz, Bruker Avance III HD Ascend 500 MHz and Bruker Avance III HD Ascend 700 MHz. ^1H NMR was reported as follows: chemical shift, multiplicity (s = singlet and d = doublet), coupling constant (*J* values) in Hz and integration. Chemical shifts (δ) were reported with respect to the corresponding solvent residual peak at 7.16 ppm. ^{13}C NMR spectra (^1H-broadband decoupled) were reported in ppm using the central peak of C_6D_6 (128.08 ppm). UV/Vis spectra were recorded with a JASCO V-750 UV-Visible Spectrophotometer at room temperature using a 10 × 10 mm quartz sample cell sealed with a J. Young valve.

2. Syntheses of Stibinidenes and Sb=Co complex

2.1. Synthesis of complex 1

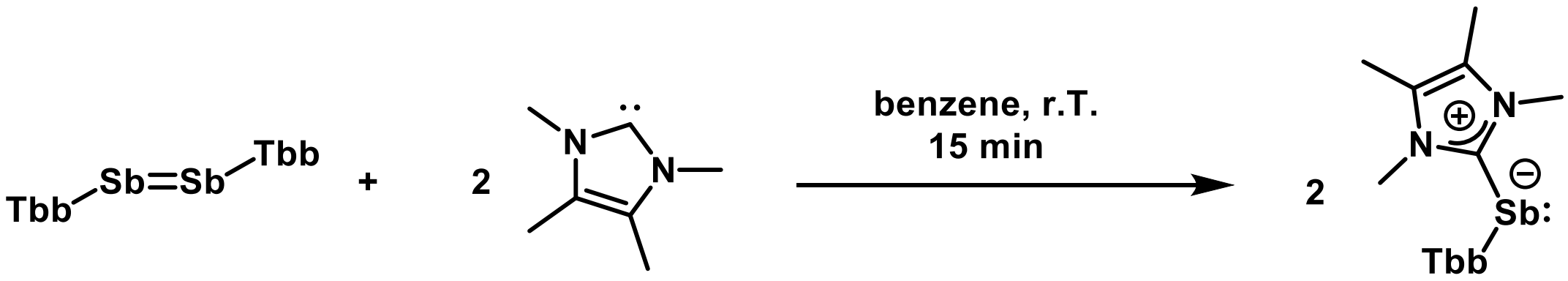

A yellow-orange suspension of Sb_2Tbb_2 (300 mg, 0.262 mmol, 1.00 equiv.) in benzene (8.0 mL) was treated with a solution of IMe_4 (67.8 mg, 0.545 mmol, 2.08 equiv.) in benzene (2.0 mL) while stirring at room temperature for 10 min. Over time, the yellow-orange suspension gradually changed into a deep red solution. Analysis of an aliquot of the reaction mixture by ^1H NMR spectroscopy confirmed the complete consumption of Sb_2Tbb_2 and selective formation of complex **1**. The solvent was evaporated under reduced pressure and the remaining solid was dried at room temperature under reduced pressure to give complex **1** as an orange-red crystalline solid (346 mg, 95% yield).

Compound **1** is an air-sensitive orange-red crystalline solid. It can be stored under argon atmosphere at –30 °C for several months. It shows very good solubility in benzene, THF, *n*-pentane and *n*-hexane at room temperature. A sample solution of **1** in C_6D_6 does not show any appreciable decomposition after 1 hour at 60 °C.

^{1}H NMR (500 MHz, C_6D_6): δ 7.04 (s, 2H), 3.90 (s, 2H), 3.25 (s, 6H), 1.43 (s, 9H), 1.36 (s, 6H), 0.24 (s, 36H).

^{13}C{^{1}H} NMR (126 MHz, C_6D_6): δ 162.9, 151.5, 147.2, 141.9, 125.1, 119.8, 38.0, 37.7, 34.2, 31.7, 8.9, 1.4.

Elemental analysis: Calcd. in % for $C_{31}H_{61}N_2SbSi_4$ (695.92 g/mol): C 53.50, H 8.84, N 4.03; found: C 53.80, H 8.85, N 4.04.

2.2. Synthesis of complex 2

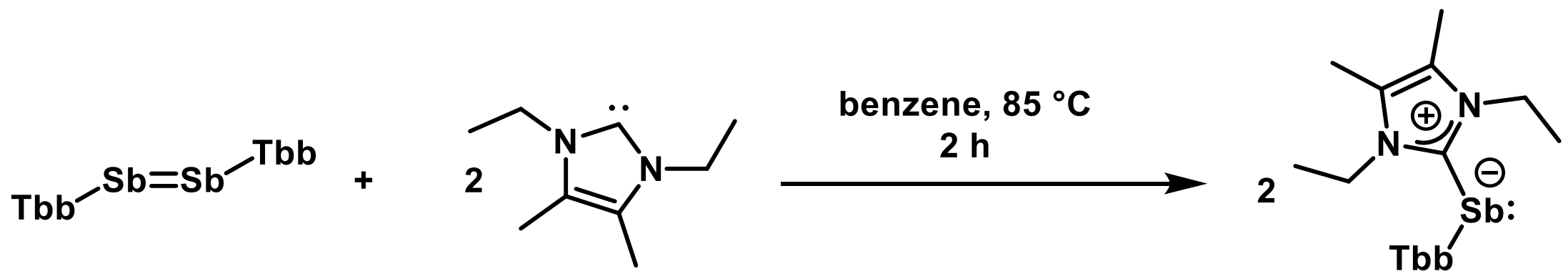

A yellow-orange suspension of Sb_2Tbb_2 (50 mg, 0.044 mmol, 1.00 equiv.) in benzene (2.0 mL) was treated with a solution of IEt_2Me_2 (13.3 mg, 0.088 mmol, 2.00 equiv.) in benzene (1.0 mL). The reaction mixture was stirred at 85 °C for 3 h. Over time, the yellow-orange suspension gradually changed into a clear red solution. Analysis of an aliquot of the reaction mixture by ^{1}H NMR spectroscopy revealed a 50% conversion of the starting material into complex **2**. Further stirring at this temperature was not improving the conversion. After cooling to room temperature, a red solution with an orange precipitate (unreacted Sb_2Tbb_2) was obtained. Filtration *via* filter cannula gave a red clear solution. The solvent was evaporated under reduced pressure giving complex **2** as a deep red crystalline solid (15 mg, 24% yield).

Note: The incomplete conversion (ca. 50%) observed for complex ***2*** *is attributed to the increased steric demand of the* IEt_2Me_2 *ligand, which establishes a thermodynamic bottleneck that remained invariant to further optimization of temperature or solvent. The low isolated yield is a consequence of the rigorous recrystallization cycles necessitated by the nearly identical solubility profiles of the product, the unreacted distibene and the NHC.*

**Solvent**	**Temperature (°C)**	**Time (h)**	**Conv (NMR)**
Benzene	85	2	47%
Toluene	110	6	50%
THF	80	2	62%

Compound **2** is an air-sensitive deep red crystalline solid. It can be stored under argon atmosphere at –30 °C for several months. It shows very good solubility in THF, benzene, *n*-hexane and *n*-pentane at room temperature.

^{1}H NMR (500 MHz, C_6D_6): δ 7.03 (s, 2H), 4.03 (q, *J* = 7.1 Hz, 4H), 3.68 (s, 2H), 1.51 (s, 6H), 1.44 (s, 9H), 1.05 (t, *J* = 7.1 Hz, 6H), 0.24 (s, 36H).

$^{13}C\{^1H\}$ NMR (126 MHz, C_6D_6): δ 159.9, 151.1, 147.0, 141.8, 125.1, 119.8, 45.4, 37.7, 34.2, 31.7, 15.5, 8.9, 1.4.

2.3. Synthesis of complex 3

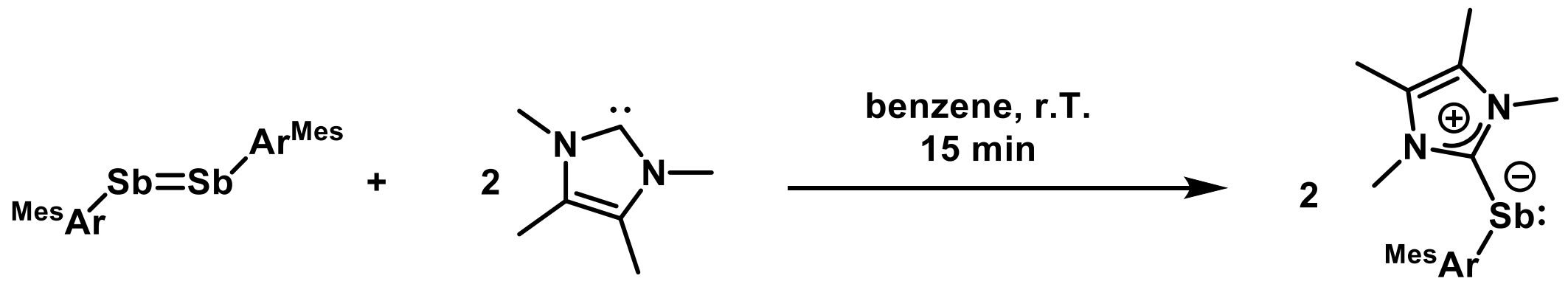

A yellow-orange suspension of $Sb_2Ar^{Mes}_2$ (100 mg, 0.115 mmol, 1.00 equiv.) in benzene (4.0 mL) was treated with a solution of IMe_4 (28.6 mg, 0.230 mmol, 2.00 equiv.) in benzene (1.0 mL) at room temperature and stirred for 10 min. Over time, the yellow-orange suspension gradually changed into a clear deep red solution. Analysis of an aliquot of the reaction mixture by ^1H NMR spectroscopy confirmed the complete consumption of Sb_2Tbb_2 and a very selective formation of complex **3**. The solvent was evaporated and the remaining solid was dried under reduced pressure to give complex **3** as an orange-red crystalline solid (120 mg, 93% yield).

Compound **3** is an air-sensitive orange-red crystalline solid. It can be stored under argon atmosphere at –30 °C for several months. It shows very good solubility in THF and benzene, but is insoluble in *n*-hexane and *n*-pentane at room temperature. A sample solution of **3** in C_6D_6 does not show any appreciable decomposition even after 1 hours at 60 °C.

^1H NMR (500 MHz, C_6D_6): δ 7.16 (t, 1H), 6.92 (d, J = 7.2, 2H), 6.72 (s, 4H), 3.17 (s, 6H), 2.47 (s, 12H), 2.11 (s, 6H), 1.25 (S, 6H).

Note: The C^4–H resonance of the central aryl ring, which typically appears as a triplet, is partially obscured by the residual solvent signal (C_6D_6) and therefore cannot be resolved unambiguously.

$^{13}C\{^1H\}$ NMR (126 MHz, C_6D_6): δ 157.3, 147.4, 146.0, 143.4, 136.1, 134.9, 128.0, 127.4, 124.2, 123.9, 39.2, 21.5, 21.3, 9.1.

Elemental analysis: Calcd. in % for $C_{31}H_{37}N_2Sb$ (559.41 g/mol): C 66.56, H 6.67, N 5.01; found: C 66.62, H 6.71, N 5.16.

2.4. Synthesis of complex 4

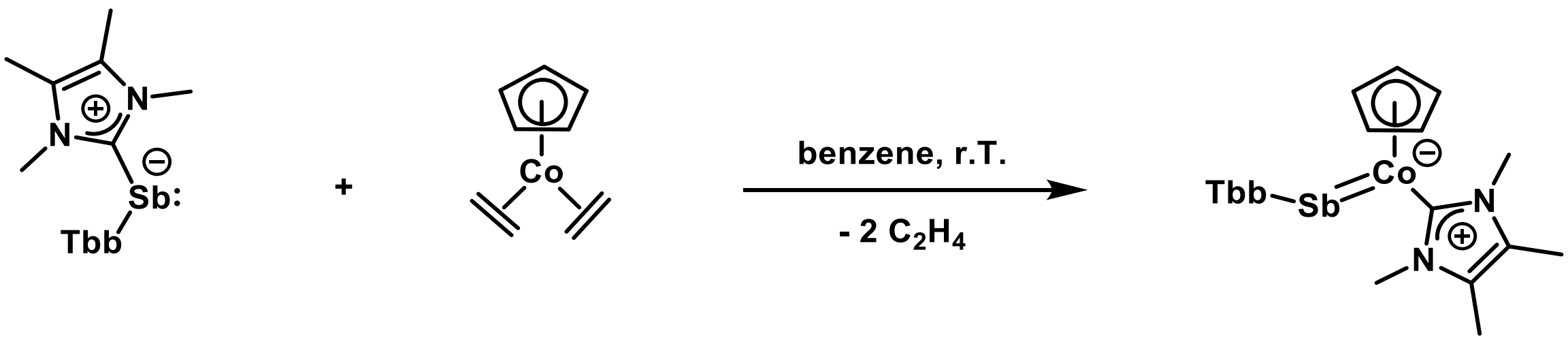

A deep red solution of complex **1** (100 mg, 0.144 mmol, 1.00 equiv.) in benzene (2.0 mL) was treated with a solution of $CpCo(C_2H_4)_2$ (28.4 mg, 0.158 mmol, 1.10 equiv.) in benzene (1.0 mL) at room temperature and stirred for 10 min. Over time, the deep red solution gradually changed into a clear brown solution. Analysis of an aliquot of the reaction mixture by ^1H NMR spectroscopy confirmed the complete consumption of complex **1** and selective formation of complex **4**. The reaction mixture was worked-up by evaporating the solvent under reduced pressure, followed by crystallization of the brown crude product from *n*-pentane (1.5 mL) at –30 °C for 16 h. Brown crystals were isolated by filtration at –30 °C and dried at room temperature under reduced pressure to give complex **4** as a brown crystalline solid (72 mg, 63% yield).

Compound **4** is a highly air-sensitive brown crystalline solid. It can be stored under argon atmosphere at –30 °C for several months. It is well soluble in benzene, THF, *n*-pentane and *n*-hexane but insoluble in MeCN at room temperature. A sample solution of **4** in C_6D_6 does not show any appreciable decomposition even after several days at room temperature and heating for several hours at 80 °C.

^{1}H NMR (500 MHz, C_6D_6): δ 7.14 (s, 2H), 4.63 (s, 5H), 3.47 (s, 6H), 2.51 (s, 2H), 1.60 (s, 6H), 1.56 (s, 9H), 0.37 (s, 36H).

^{13}C{^{1}H} NMR (126 MHz, C_6D_6): δ 187.0, 159.7, 149.0, 147.7, 126.2, 121.8, 74.8, 36.9, 34.4, 32.0, 31.7, 9.0, 1.6.

Elemental analysis: Calcd. in % for $C_{36}H_{66}CoN_2SbSi_4$ (819.97 g/mol): C 52.73, H 8.11, N 3.42; found: C 53.80, H 8.66, N 3.37.

2.5. Synthesis of complex 5

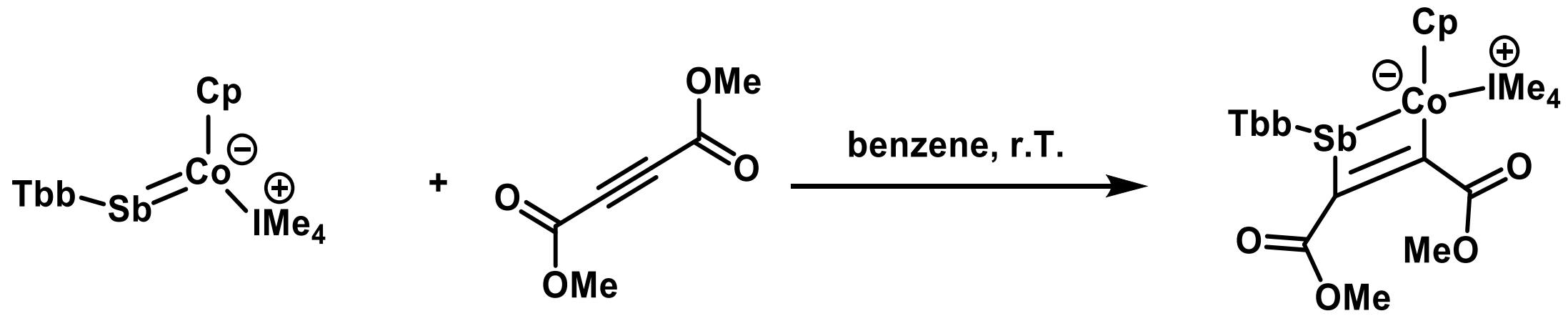

A brown solution of complex **4** (30 mg, 0.037 mmol, 1.00 equiv.) in benzene (1.5 mL) was treated with a solution of dimethylacetylenedicarboxylate (5.2 mg, 0.037 mmol, 1.00 equiv.) in benzene (0.5 mL) at room temperature and stirred for 1.5 h. Over time, the brown solution gradually changed into a dark red solution. Analysis of an aliquot of the reaction mixture by ^{1}H NMR spectroscopy confirmed the complete consumption of complex **4** and selective formation of complex **5**. The reaction mixture was worked-up by evaporating the solvent under reduced pressure, followed by washing with cold *n*-pentane (1 x 0.5 mL). The obtained grey-red crystalline solid was dried under reduced pressure, giving complex **5** (22 mg, 62%) in analytically pure form.

Compound **5** is a highly air-sensitive grey-red solid. It can be stored under argon atmosphere at –30 °C for at least one week. It is well soluble in benzene and THF, but insoluble in *n*-pentane.

^{1}H NMR (500 MHz, C_6D_6): δ 6.94 (s, 2H), 4.73 (s, 5H), 3.71 (s, 3H), 3.68 (br s, $\Delta\nu_{1/2}$ = 47 Hz, 6H), 3.44 (s, 3H), 2.64 (s, 2H), 1.42 (s, 9H), 1.31 (s, 6H), 0.43 (s, 18H), 0.37 (s, 18H).

^{13}C{^{1}H} NMR (126 MHz, C_6D_6): δ 174.4, 173.9, 160.7, 157.6, 149.5, 147.0, 143.0, 128.7, 126.5, 123.3, 83.0, 50.4, 50.3, 37.6, 34.0, 31.0, 30.0, 8.7, 1.4, 1.3.

2.6. Synthesis of complex 6

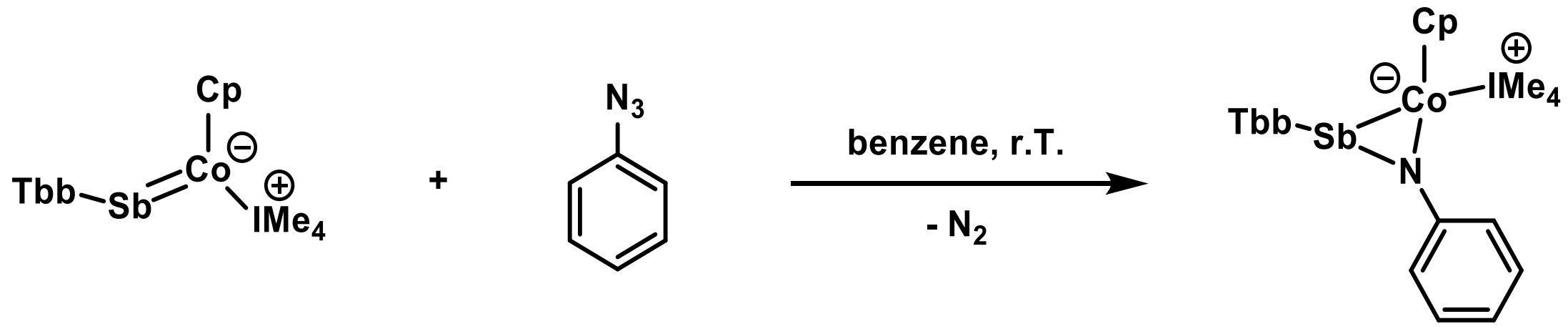

A brown solution of complex **4** (25 mg, 0.030 mmol, 1.00 equiv.) in benzene (1.5 mL) was treated with a solution of phenylazide (0.06 mL, 0.030 mmol, 1.00 equiv.) in MTBE (0.5 M) at room temperature and stirred for 30 min. Over time, the brown solution gradually changed into a black-brown solution. Analysis of an aliquot of the reaction mixture by ^{1}H NMR spectroscopy confirmed the complete consumption of complex **4** and selective formation of complex **6**. The reaction mixture was worked-up by evaporating the solvent under reduced pressure, followed by crystallization of the black-brown crude product from *n*-hexane (0.5 mL) at –30 °C for 16 h. Black-brown crystals were isolated by filtration at –30 °C and dried at room temperature under reduced pressure to give complex **6** as a black-brown crystalline solid with 25% impurity of oxygen-bridged distibane complex **7** (10 mg, 36% yield).

Compound **6** is a highly air-sensitive black-brown crystalline solid. It is moderately soluble in benzene, THF, *n*-pentane and *n*-hexane.

Note: Unfortunately, complex ***6*** *is highly unstable and a formation of a side product was observed during the reaction and the characterization. Due to same solubility any attempt (washing / crystallization) of removing the side product failed. However, the side product could be identified as oxygen-bridged distibane (complex* ***7****) by X-ray analysis and ^{1}H NMR spectroscopy.*

^{1}H NMR (500 MHz, C_6D_6): δ 7.08 (m, 4H), 6.97 (s, 2H), 6.85 (m, 1H), 4.81 (s, 5H), 3.95 (bs, 3H), 3.63 (s, 2H), 3.19 (bs, 3H), 1.41 (s, 9H), 1.23 (bs, 6H), 0.36 (s, 36H).

^{13}C{^{1}H} NMR (126 MHz, C_6D_6): δ 166.4, 150.0, 148.3, 147.2, 128.8, 125.9, 122.7, 116.6, 81.1, 34.4, 31.5, 29.9, 8.8, 1.6.

3. UV/Vis Spectroscopic Studies

All UV/vis measurements were conducted according to the standard operating procedure reported below.

All stock solutions were prepared in an Argon filled glovebox and immediately diluted and placed in a cuvette sealed with a J. Young valve. UV/Vis-measurements were carried out in *n*-hexane at room temperature. All spectra were recorded in a range from 700-300 nm in data intervals of 1 nm. The UV/Vis bandwidth was set to 1.0 nm.

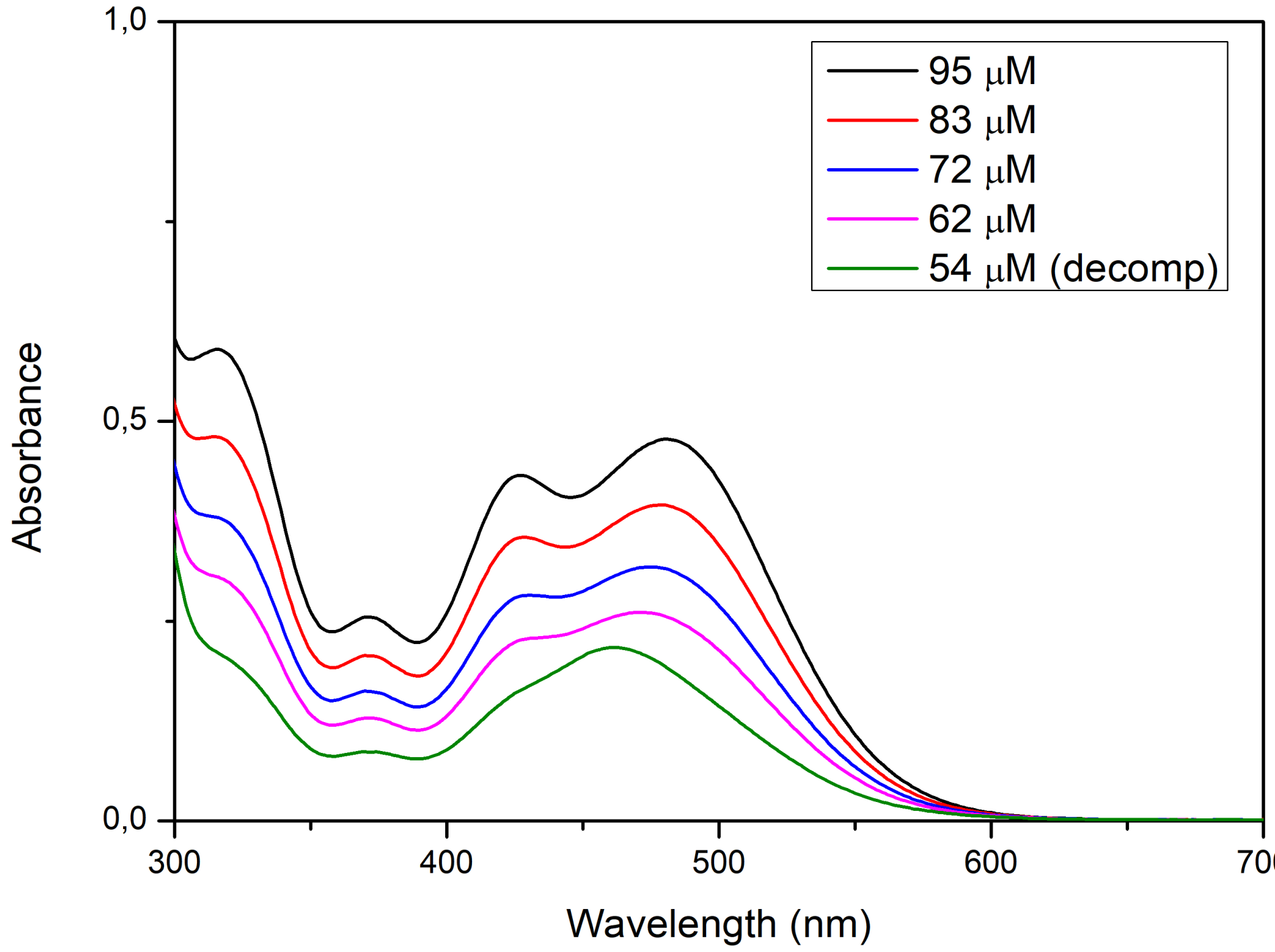

Figure S1. UV/Vis spectra of complex **1** at different concentrations in *n*-hexane at room temperature.

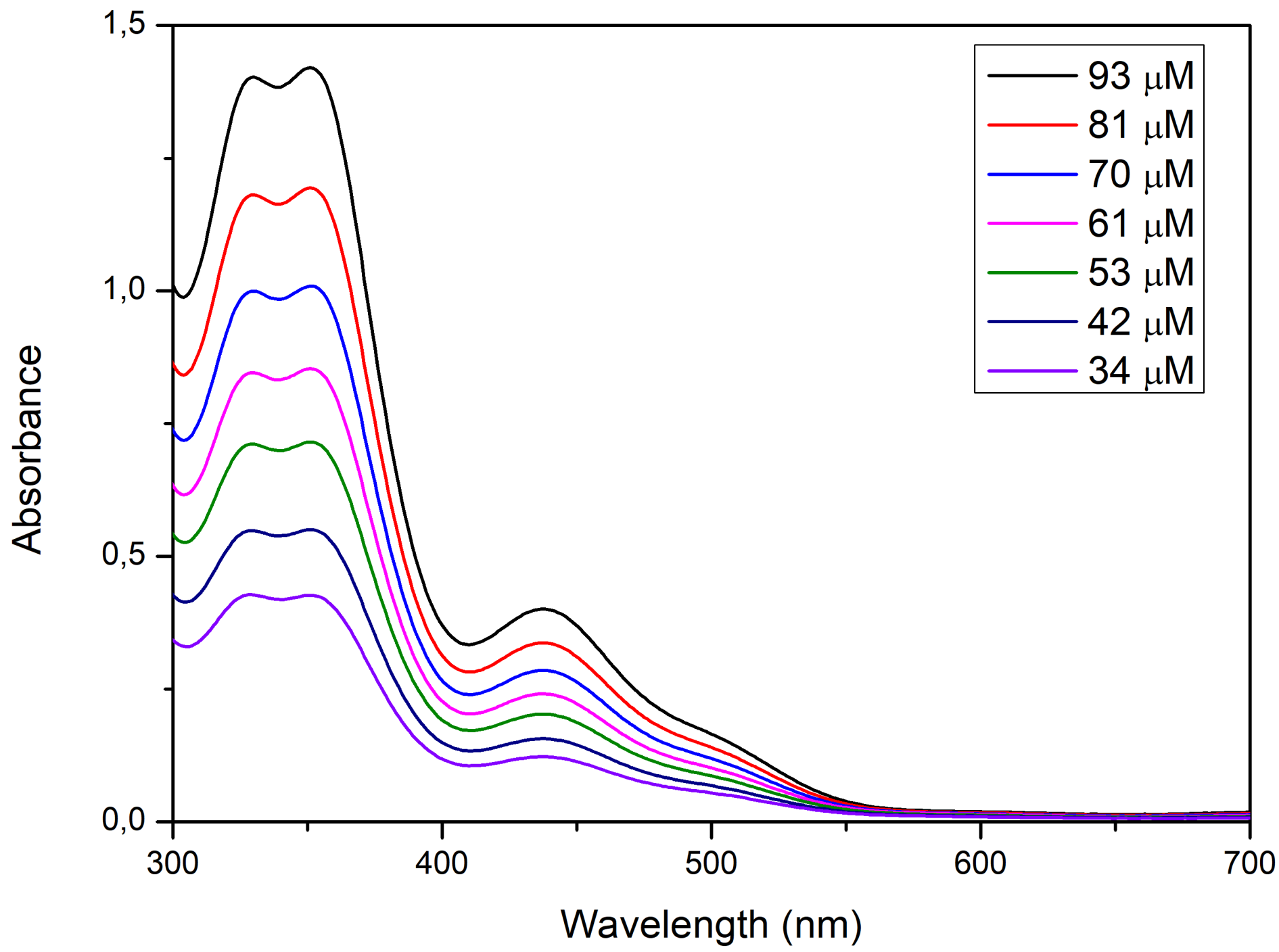

Figure S2. UV/Vis spectra of complex **4** at different concentrations in *n*-hexane at room temperature.

4. Electrochemical Studies

Cyclic voltammetry (CV) was performed using a Palmsens4 potentiostat with all CVs being run in a glovebox insuring a moisture and O_2-free environment. A homogeneous system was employed using glassy carbon as the working electrode, Pt wire as counter electrode and an $Ag/AgNO_3$ organic reference electrode in a one-compartment cell. These measurements were carried out in dry THF with 0.1 M $TBAPF_6$ which had been dried in an oven to remove any moisture. 1 mM of the complex was used scanning in the range of 1.0 ~ –1.0 V (vs. $Ag/AgNO_3$) at a sweep rate of 100 mV s^{-1}.

All CV measurements were conducted using a non-aqueous silver/silver nitrate ($Ag/AgNO_3$) reference electrode (0.01 M $AgNO_3$ in acetonitrile with 0.1 M tetrabutylammonium hexafluorophosphate). To ensure the stability and accuracy of the reference potential, ferrocene (Fc) was used as an internal standard in separate measurements before and after each experimental series to ensure no potential drift.

Cyclic voltammetry of complex **4** (see Figure S3) in THF (0.1 M $TBAPF_6$) shows a quasi-reversible redox couple at –0.044 V (oxidation) and –0.34 V (reduction), accompanied by an additional irreversible oxidation at –0.46 V vs Fc/Fc^+. The quasi-reversible nature of the first redox couple, with a modest separation between oxidation and reduction peaks, indicates that the electron transfer at the cobalt center is relatively stable, although some structural or electronic reorganization likely occurs. Comparison with $CpCo(C_2H_4)_2$, which exhibits a similar pattern of two oxidative and one reductive feature under the same conditions (Figure S4), reinforces the assignment of a Co(I)-like resting state for the complex. In both cases, the first quasi-reversible couple, along with the overall similarity in redox behavior to $CpCo(C_2H_4)_2$, is consistent with a low-valent, electron-rich cobalt(I) resting state.

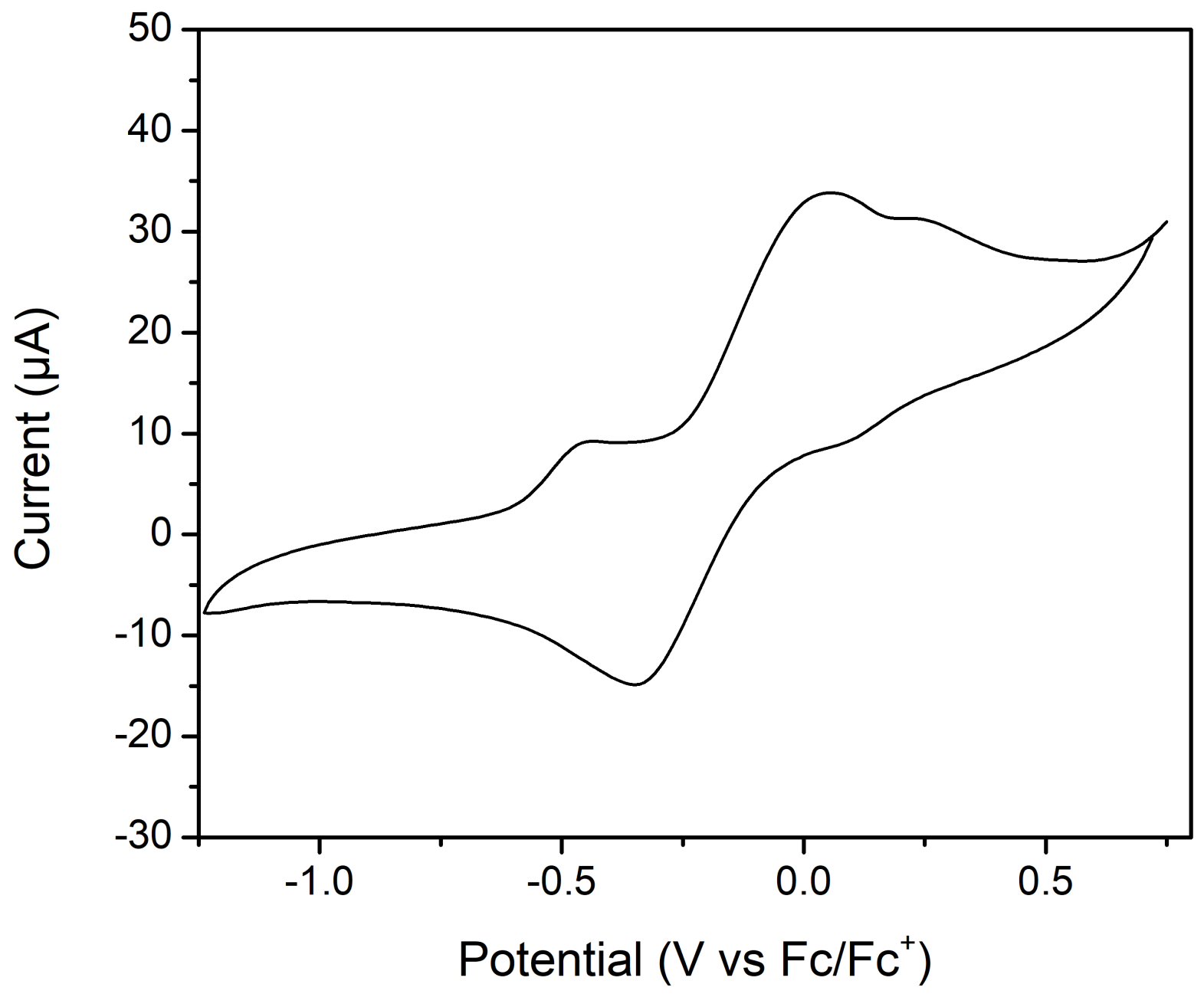

***Figure S3*.** Cyclic voltammogram of complex **4** in THF with 0.1M $TBAPF_6$ supporting electrolyte at 100mV s^{-1}.

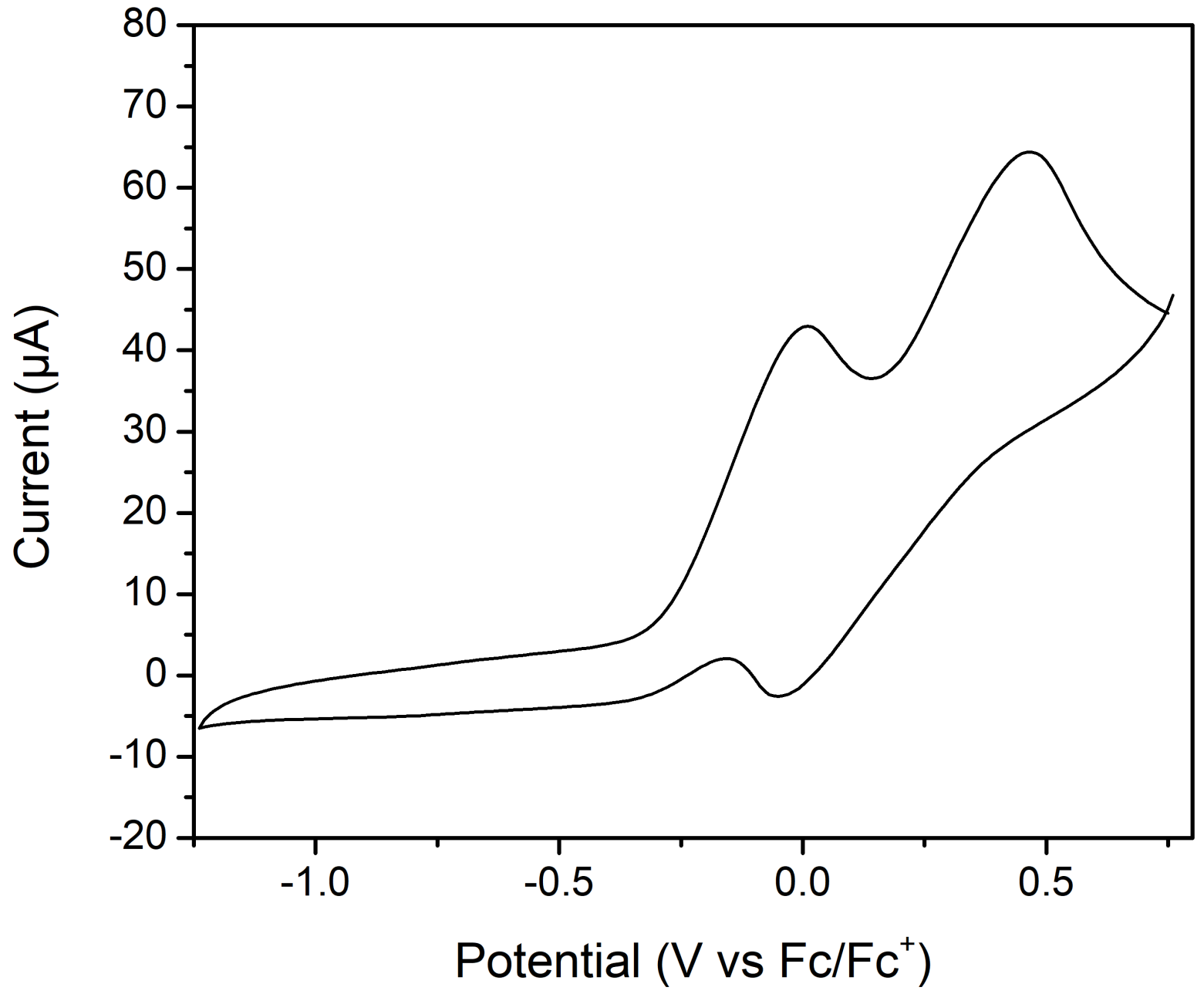

***Figure S4*.** Cyclic voltammogram of $CpCo(C_2H_4)_2$ in THF with 0.1M $TBAPF_6$ supporting electrolyte at 100mV s^{-1} showing only irreversible behaviour.

4.1. Scan Rate Dependence of Complex 4

To investigate the quasi-reversible oxidation process observed for complex **4**, cyclic voltammograms were recorded at scan rates ranging from 25 to 500 mV s^{-1} (Figure S5a). A systematic increase in peak-to-peak separation (ΔE) was observed with increasing scan rate, increasing from 0.20 V at 25 mV s^{-1} to 0.71 V at 500 mV s^{-1} (Figure S5b). Such behaviour is characteristic of a kinetically quasi-reversible electron-transfer process and indicates significant deviation from ideal reversible electrochemical behaviour.

In addition to the increase in ΔE, the ratio of the reductive to oxidative peak currents (I_{pc}/I_{pa}) increased from 0.29 at 25 mV s^{-1} to approximately 0.60 at 500 mV s^{-1} (Figure S5c). The enhanced recovery of the oxidized species at higher scan rates suggests that the oxidized form of **4** is more effectively reduced on shorter experimental timescales. This behaviour is consistent with electronic and/or structural reorganization following oxidation, which increasingly competes with the reverse electron-transfer process at lower scan rates.

To further evaluate the electrochemical behaviour, ΔE was plotted as a function of log(scan rate) (Figure S5d). A linear relationship was observed across the investigated scan-rate range, further supporting the assignment of a kinetically quasi-reversible redox process.

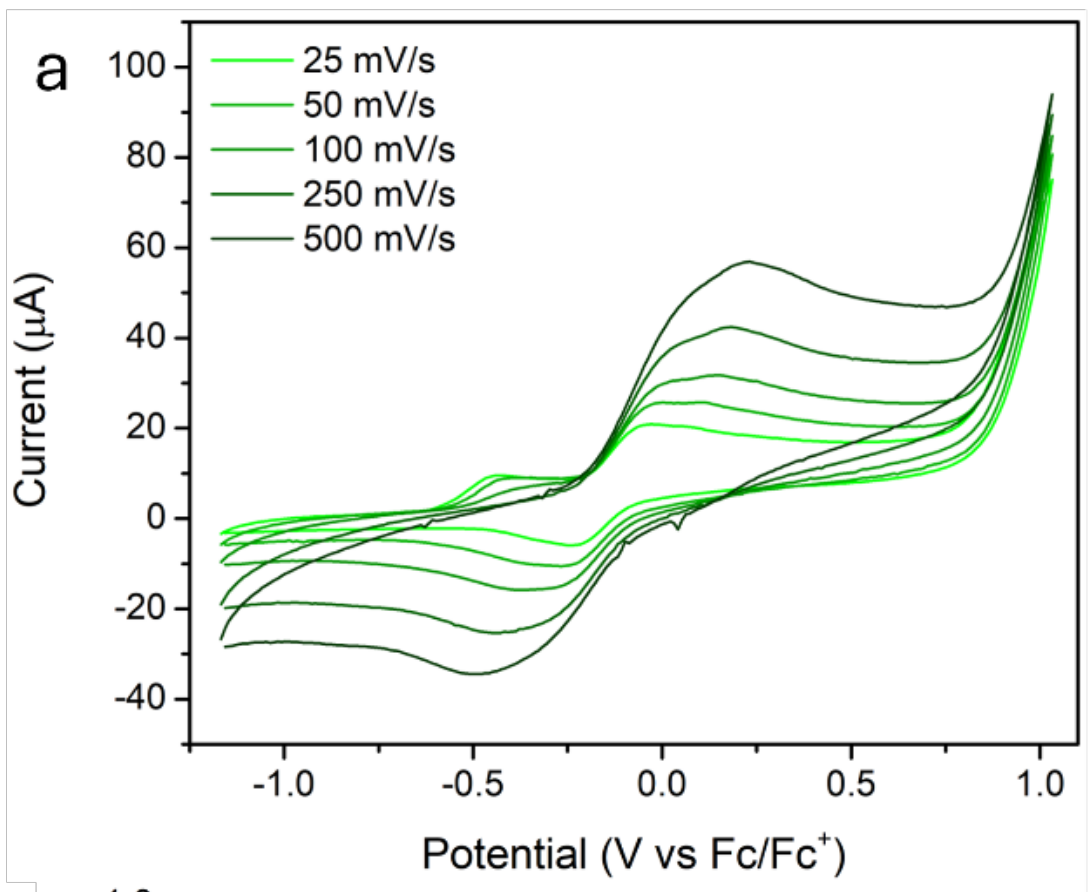

b

Scan Rate (mV/s)	I_{pc}/I_{pa}	ΔE (V)
25	0.29	0.2
50	0.42	0.32
100	0.49	0.50
250	0.59	0.62
500	0.60	0.71

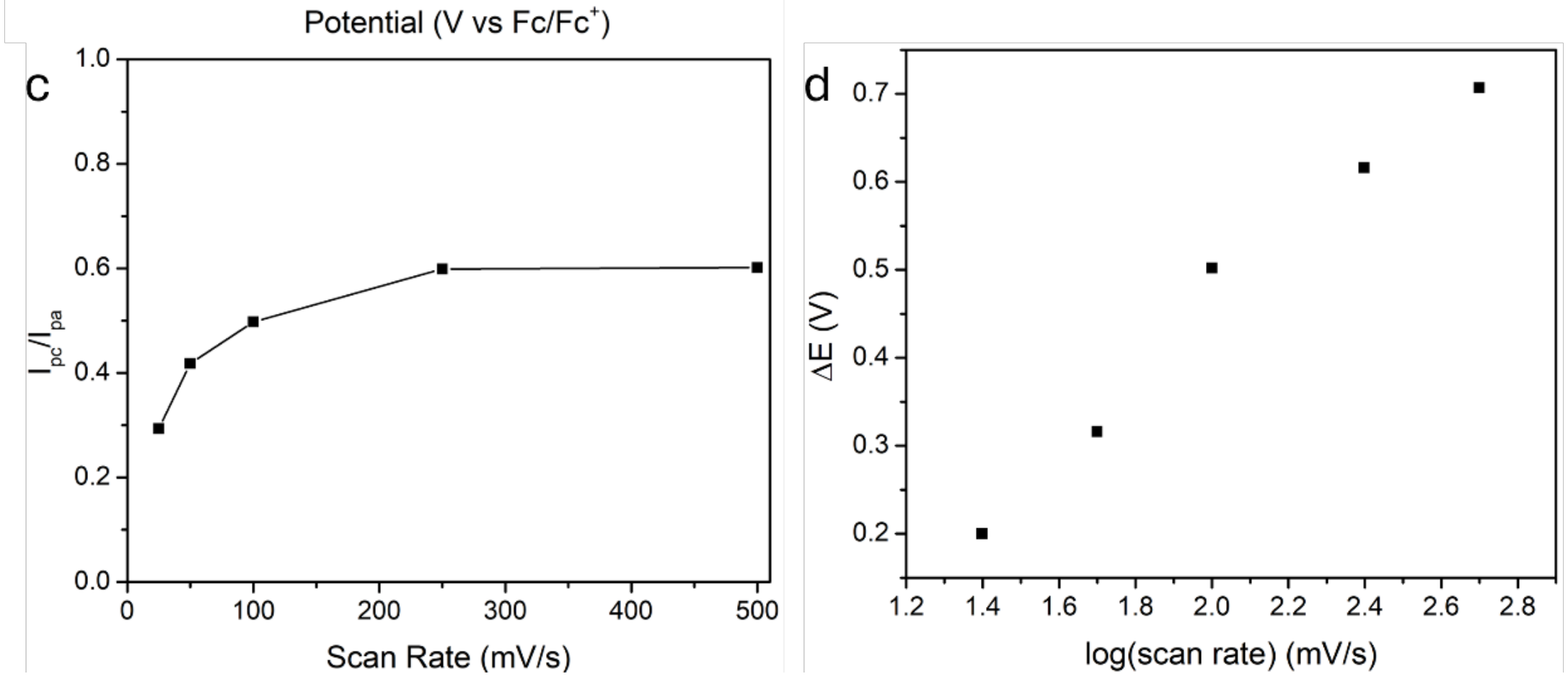

Figure S5. Variable scan rate cyclic voltammetry studies of complex **4**. (a) Overlayed cyclic voltammograms recorded at various scan rates. (b) Summary of electrochemical parameters obtained from the voltammograms, including peak-to-peak separation (ΔE) and the ratio of reductive to oxidative peak currents (I_{pa}/I_{pc}). (c) Dependence of I_{pa}/I_{pc} on scan rate. (d) Plot of ΔE versus log(scan rate).

5. Computational methods and DFT calculations

5.1. Computational methods

Geometry optimizations without symmetry constraints were performed at the TPSS-D4/def2-TZVP level of theory[5-8] using the Turbomole 7.7 program[9]. For Sb atoms, effective core potentials (ECPs) were employed with inclusion of relativistic effects in the basis set definition.[7, 8] This level of theory has been successfully applied to similar systems.[1, 10, 11]

Theoretical analysis was conducted using the ORCA 6.0.1 software.[12] Single point energies were computed using the double hybrid PW6B95 using the 'exact two component' (X2C) Hamiltonian[13] module implemented in ORCA in combination with the dispersion correction term D4, as a basis set x2c-QZVPPall was chosen.[14] For cost efficient calculations the RI approximation[15] was applied using automatic selected auxiliary basis sets.[16] The X2C treatment and the x2c-QZVPPall basis set were chosen to address the relativistic effects for all electrons. Localised orbitals, *e.g.*, intrinsic bond orbitals (IBO),[17] were also computed on the same level. The natural bond orbital (NBO) and the Natural Localized Molecular Orbital (NLMO) analyses were computed using the NBO 7.0 program.[18] The NBO 7.0 calculations were performed at the x2c-PW6B95-D4/x2c-QZVPPall/TPSSD4/def2-TZVP level of theory. Mayer bond order[19] and Wiberg bond indices[20] and ETS-NOCV[21] were computed using MultiWFN[22] at the same level of theory. Orbitals were visualised with Gaussview 6.0.[23] Orbital surfaces were plotted with an isosurface value of 0.04 a.u. TD-DFT calculations were performed at the TPSS-D4/def2-TZVP level of theory in hexane and considering 30 excited states using ORCA 6.0.1 program. Other functionals like wB97D and CAM-B3LYP were also tested, but a worse agreement with the experimental spectra of **1** and **4** was observed.

5.2. DFT calculations

Figure S6 presents the optimized structures of compounds **1**–**3**, using the X-ray geometries as starting points (TPPS-D4/def2-TZVP level of theory). This functional has been demonstrated to be effective for studying main group elements. A good correlation between the experimental and theoretical geometries is observed, with very similar bond lengths. For instance, the X-ray Sb–C1 bond length is 2.192(4) Å and the Sb(1)–C^{NHC} bond distance 2.171(4) Å in **2**. This consistency reinforces the reliability of the chosen functional and basis set used in this work.

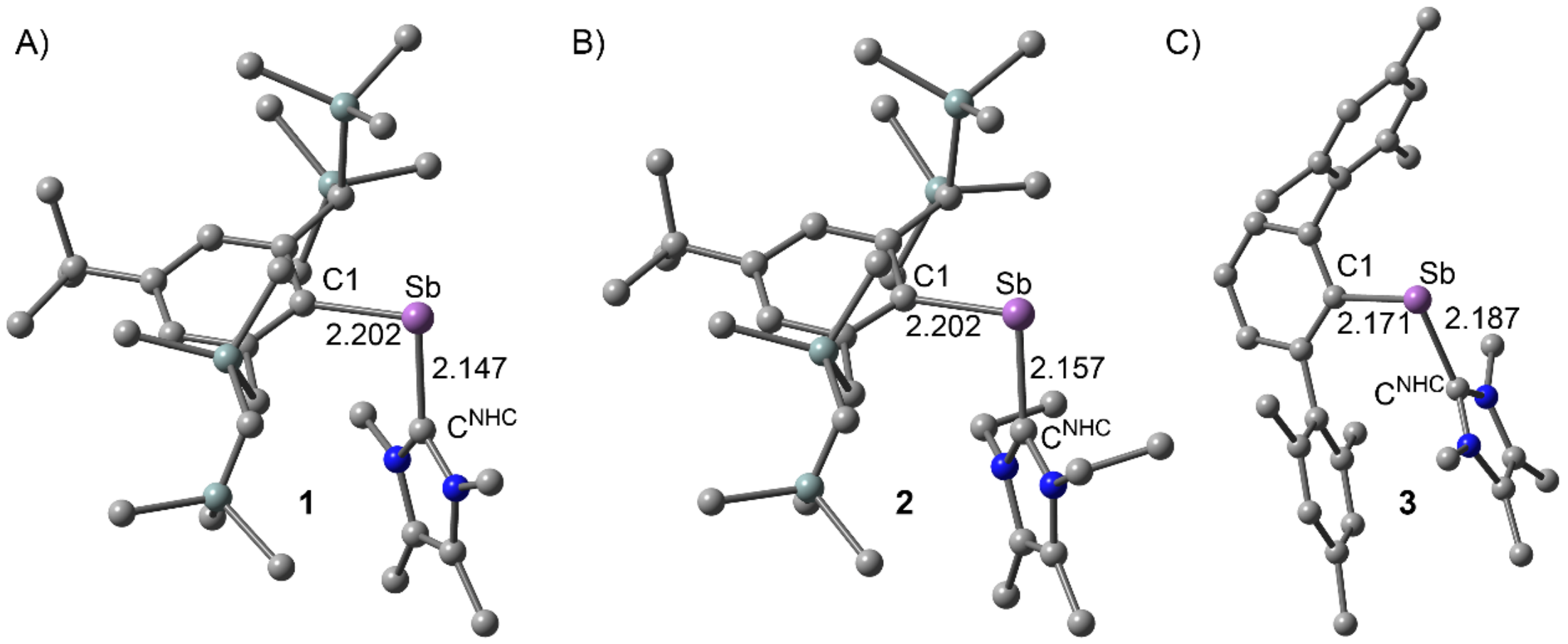

Figure S6. Optimized geometries of compounds **1** (A), **2** (B) and **3** (C). Distances in Å.

Analysis of the frontier molecular orbitals (Figure S7) reveals that the HOMO corresponds to the lone pair on the antimony atom, while the LUMO is localized on the NHC framework and partially delocalized to the Ar^{Mes} in compound **3**. This electronic topography aligns with the expected nucleophilicity of the stibinidene motif. The Wiberg Bond Indices (WBI) and Mayer Bond Orders (MBO) are close to unity (approx. 1.0 and 1.3, respectively), which effectively rules out the formation of a C=Sb double bond. Instead, these metrics support the zwitterionic resonance structure depicted in Figure S7B&D (NHC^+–Sb^-).

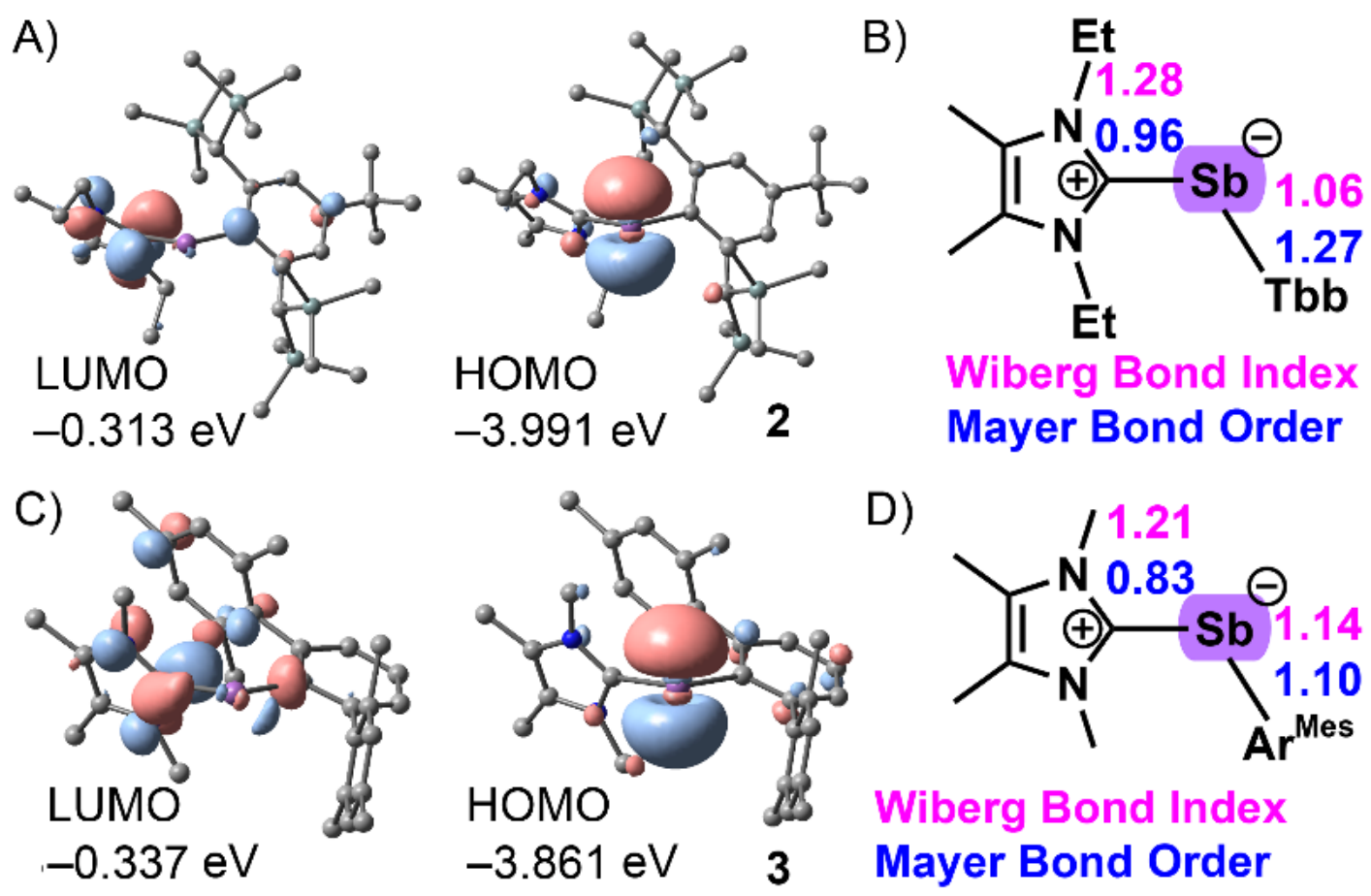

Figure S7. FMOs of complexes **2** (A) and **3** (C) and Wiberg bond index (pink) and Mayer bond order (blue) values for both Sb–C bonds (B, D) at the x2c-PW6B95-D4/x2c-QZVPPall//TPSS-D4/def2-TZVP level of theory.

Figure S8 shows the NBO corresponding to the σ-orbital of the Sb–C^{NHC} bond in compound **1**.

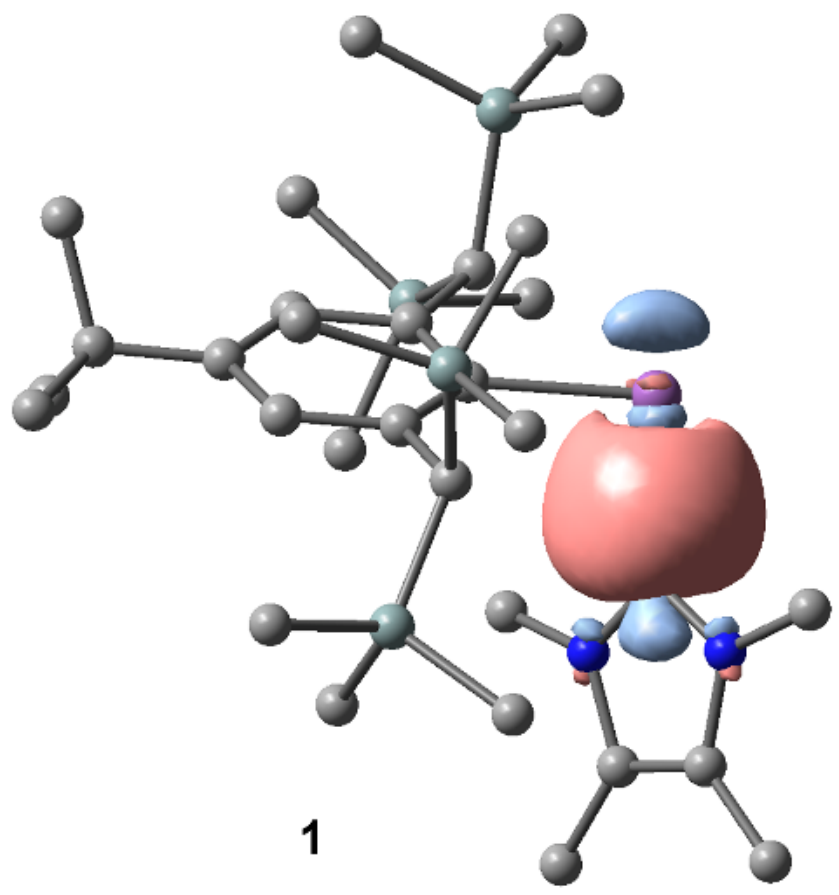

Figure S8. Natural Bond Orbitals (NBO) depicting the σ-orbital of the Sb–C^{NHC} bond in compound **1** at the x2c-PW6B95-D4/x2c-QZVPPall//TPSS-D4/def2-TZVP level.

An analysis of the Co–Sb bond in compound **4** was performed. As shown in Figure S9, the optimized structure exhibits a Co–Sb bond length shorter than the experimentally determined distance of 2.407 Å. This shortening is likely a consequence of the thermal disorder observed in the solid-state X-ray structure.

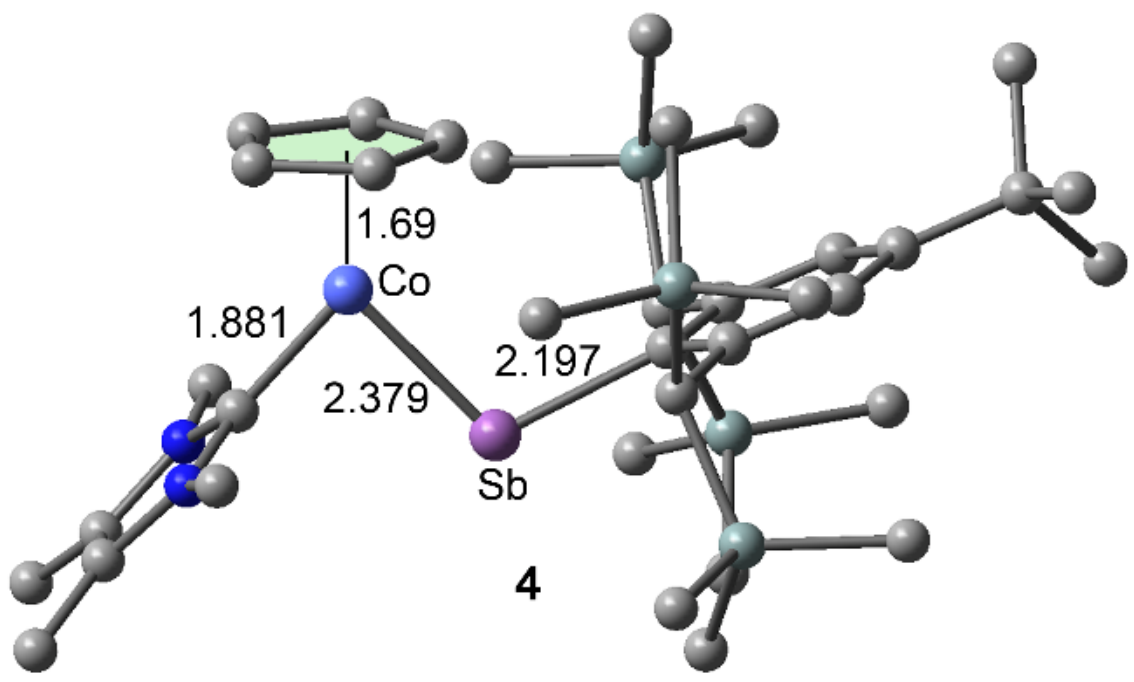

Figure S9. Geometry of **4** at the x2c-PW6B95-D4/x2c-QZVPPall /TPSS-D4/def2-TZVP level of theory.

The Intrinsic Bond Orbitals (IBOs), presented in Figure S10 from LUMO+1 to HOMO–5, are highly similar to the localized orbitals. It is noteworthy that the highest occupied IBO and IBO–1 correspond closely to the NBOs shown in Figure 4 (main text). The spatial distribution of the frontier molecular orbitals (LUMO, HOMO, and HOMO–1) across the Sb=Co bond anticipates that the compound's reactivity will be centered at this site.

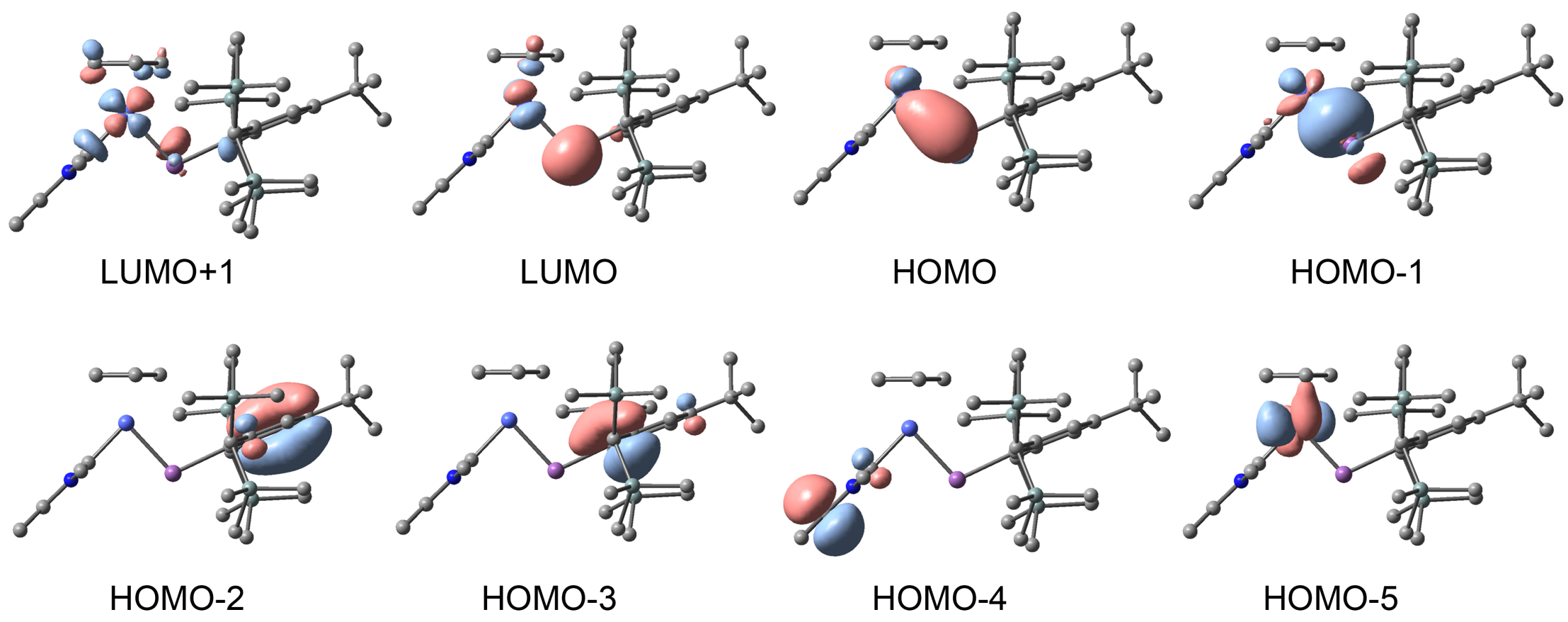

***Figure S10*.** IBOs at the x2c-PW6B95-D4/x2c-QZVPPall /TPSS-D4/def2-TZVP level of theory for compound **4**.

The plots of the Kohn–Sham molecular orbitals are provided in Figure S11, which depicts the HOMO, HOMO–1, and LUMO. Subsequent orbitals are localized on the aromatic ring of the Tbb ligand and are therefore less informative for predicting reactivity. The LUMO corresponds to the π* orbital of the Co=Sb bond, while the HOMO and HOMO–1 represent the bonding π and σ orbitals, respectively. Therefore, in agreement with the IBO analysis, the reactivity is expected to be centered on this bond.

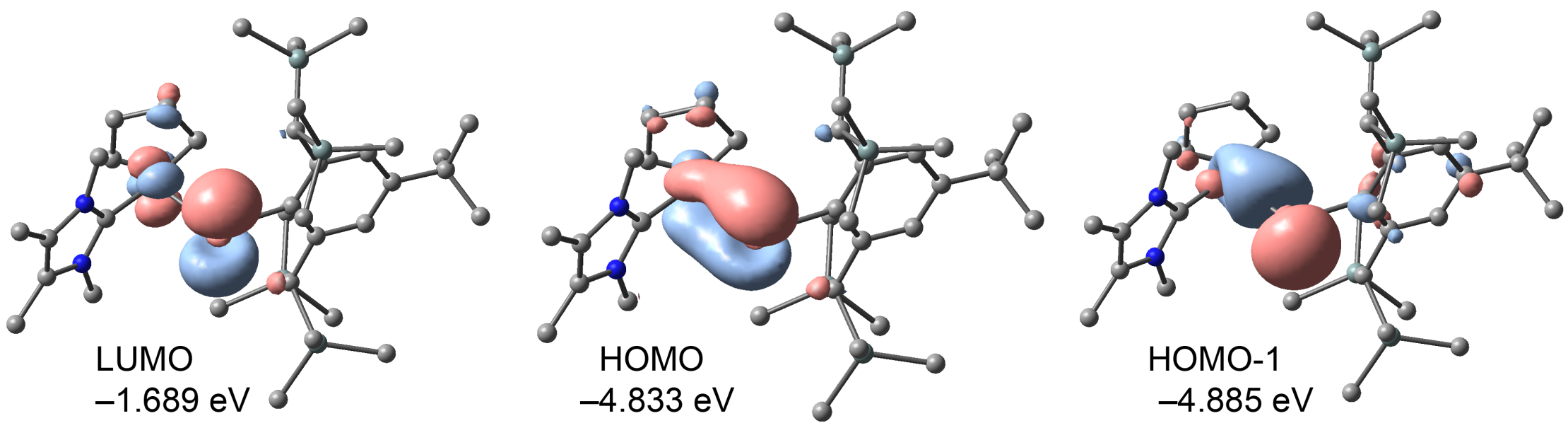

***Figure S11*.** Kohn–Sham MOs and related energies at the x2c-PW6B95-D4/x2c-QZVPPall /TPSS-D4/def2-TZVP level of theory.

To confirm the covalent double-bond character of the Co=Sb interaction and rule out a major dominance of electrostatic effects, an ETS-NOCV analysis was carried out. This analysis identifies two main deformation density pairs, which are summarized in Table S1, along with a total orbital stabilization energy of –110.03 kcal/mol and a total eigenvalue (electron sharing) of 4.16 e. These values are in excellent agreement with the presence of a Co=Sb double bond. The detailed composition, orbital energies, and eigenvalues for the two primary contributors are listed in Table S1. Specifically, the eigenvalues for pairs 1 and 2 are 1.80 and 0.51, respectively, which clearly rules out an electrostatically dominated interaction where electron

sharing would be negligible. Furthermore, the relative contributions of the Sb and Co atomic orbitals to these two primary NOCV pairs are in good agreement with the NBO data discussed in the main text.

Table S1. ETS-NOCV results for the two main pairs for the Co=Sb linkage in compound **4**.

Pair	composition of Sb &Co	Energy (kcal/mol)	Eigenvalue (a.u.)
1	61% Sb 39%Co	–78.4	1.80
2	42% Sb 58 % Co	–15.7	0.51

TD-DFT calculations were performed to assign the observed electronic transitions for compounds **1** and **4**. The calculated spectra are in good agreement with the experimental data shown in Figure S12. The calculated excitation energies, theoretical absorption bands, and detailed orbital compositions for the transitions are summarized in Table S2.

For compound **1**, the broad experimental absorption band around 480 nm consists of two electronic excitations ($S_0 \rightarrow S_1$ and $S_0 \rightarrow S_2$) with similar molecular orbital contributions. Both excitations primarily involve transitions from the HOMO to the LUMO and LUMO+1 in comparable proportions. For the $S_0 \rightarrow S_2$ excitation, a minor contribution from the HOMO → LUMO+2 transition (18.4 %) is also observed. The band centered at 425 nm corresponds to the $S_0 \rightarrow S_3$ excitation and is heavily dominated by the HOMO → LUMO+1 transition (80.0 %). Finally, the higher-energy band at 362 nm is assigned to the $S_0 \rightarrow S_5$ excitation, which is composed entirely of a single HOMO → LUMO+4 transition. The molecular orbitals involved in these transitions are depicted in Figure S13, revealing a predominant lone pair to π* (LP → π*) character. Specifically, the HOMO corresponds to the lone pair located on the Sb atom, whereas the antibonding π* orbitals are distributed over the Tbb ligand and the NHC ring.

For compound **4**, the lowest-energy calculated band appears at 703.7 nm (f = 0.0194). However, the calculated oscillator strength is somewhat overestimated compared to the experimental spectrum, which exhibits very low absorbance around 700 nm. This band corresponds to the $S_0 \rightarrow S_2$ excitation and is dominated by the HOMO → LUMO transition (70.4 %). The next prominent excitation is predicted at 445 nm, matching the experimental absorption band well. This feature is assigned to the $S_0 \rightarrow S_7$ excitation, which comprises a mixed character of 48.5 % HOMO-3 → LUMO, 11.4 % HOMO-2 → LUMO+1, and 23.7 % HOMO-1 → LUMO+1. The absorption band at 388 nm is attributed to the S0 → S10 excitation,

with the primary contributions arising from the HOMO-4 → LUMO+1 (17.8 %) and HOMO-1 → LUMO+1 (52.9 %) transitions. The molecular orbitals involved in these transitions are displayed in Figures S11 and S13, showing a predominant π → π* character with a moderate degree of metal-to-ligand charge transfer (d → π*) character.

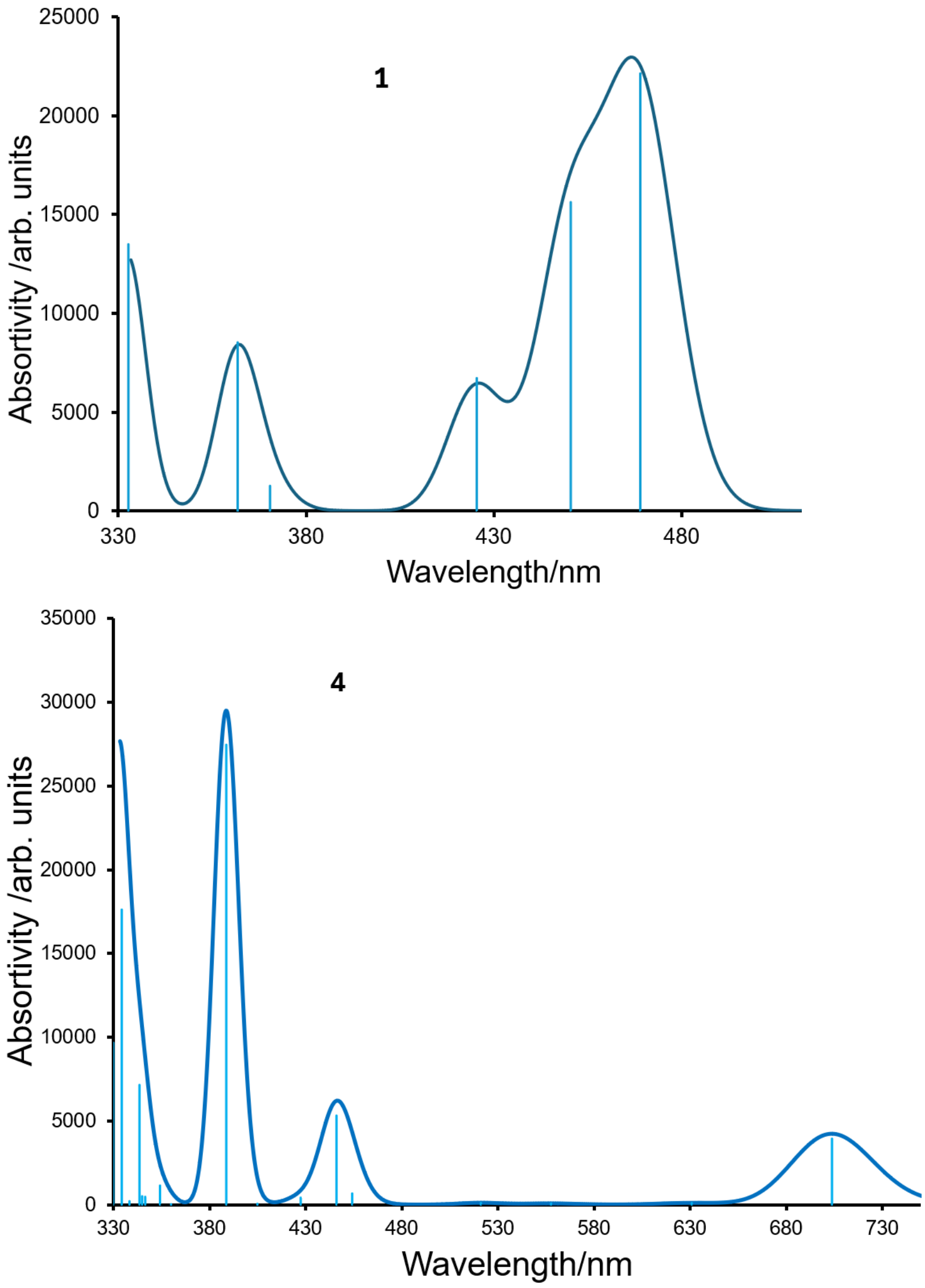

Figure S12. Theoretical UV/vis spectra of **1** (top) and **4** (bottom).

Table S2. TD-DFT results for the absorption spectra of compounds **1** and **4**.

Excitation	λ (nm)	f	Composition (>10%)
Compound **1**			
$S_0 \rightarrow S_1$	469	0.0956	48% HOMO → LUMO<br>49.8% HOMO → LUMO+1
$S_0 \rightarrow S_2$	450.5	0.0675	36.5% HOMO → LUMO<br>42% HOMO → LUMO+1<br>18.4% HOMO → LUMO+2
$S_0 \rightarrow S_3$	425.4	0.0290	10.3% HOMO → LUMO<br>80.6% HOMO → LUMO+1
$S_0 \rightarrow S_5$	361.7	0.0369	93.4% HOMO → LUMO+4
Compound **4**			
$S_0 \rightarrow S_2$	703.7	0.0194	21.9%HOMO–3 → LUMO<br>70.4% HOMO → LUMO
$S_0 \rightarrow S_7$	445.9	0.0263	48.5% HOMO–3 → LUMO<br>11.4% HOMO–2 → LUMO+1<br>23.7% HOMO–1 → LUMO+1
$S_0 \rightarrow S_{10}$	388.6	0.1357	17.8% HOMO–4 → LUMO+1<br>52.9% HOMO–1 → LUMO+1

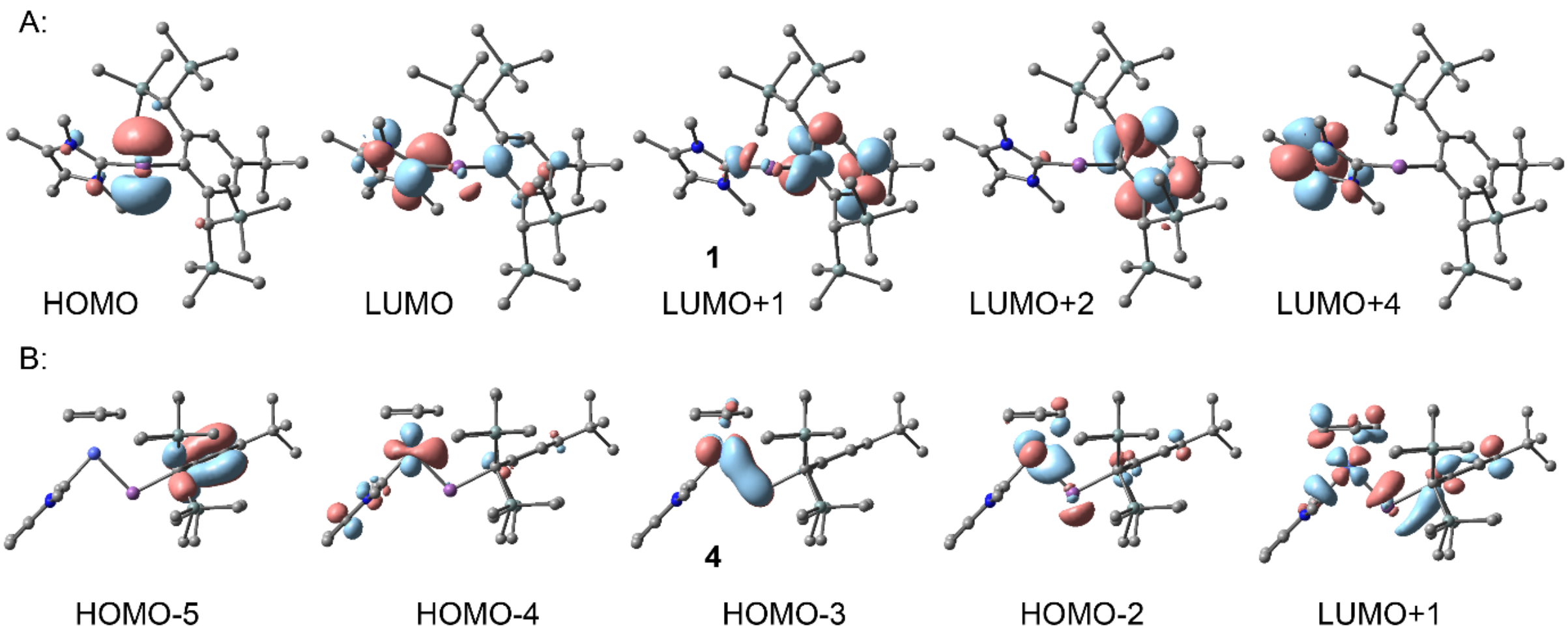

Figure S13. MOs of compound **1** (A) and **4** (B) involved in the excitations described in Table S2.

We have also investigated the migration of the IMe_4 ring from the Sb atom to the Co center during the formation of compound **4** from **1** using DFT calculations. For this study, we employed a simplified model where the Tbb ligand was replaced by a less bulky group to reduce computational cost and facilitate the transition state (TS) location by minimizing the number of methyl groups (see Figure S14A). Compounds with multiple methyl groups frequently exhibit spurious imaginary frequencies corresponding to low-energy methyl rotations, which significantly complicates the TS search and its subsequent characterization.

We propose a plausible mechanism for the formation of **4'** (the reduced model of **4**) from **1'** (the reduced model of **1**). The process begins with a ligand substitution step, where compound **1'** replaces a coordinated ethene ligand from the $CpCo(C_2H_4)_2$ complex to yield the intermediate (INT, see Figure S14B). This initial step is exergonic by –4.5 kcal/mol in terms of solution Gibbs free energies computed at the TPSS-D4/def2-TZVP level of theory. Subsequently, the migration of the IMe_4 ring takes place with the concomitant dissociation of the second ethene ligand. This alkene elimination occurs at an early stage of the migration path and is not involved in the actual transition state (see Figure S14C). The calculated activation barrier for this step is only 7.8 kcal/mol, which is in excellent agreement with the mild experimental conditions required for the reaction. Overall, the entire transformation is thermodynamically favorable and exergonic by -10.3 kcal/mol.

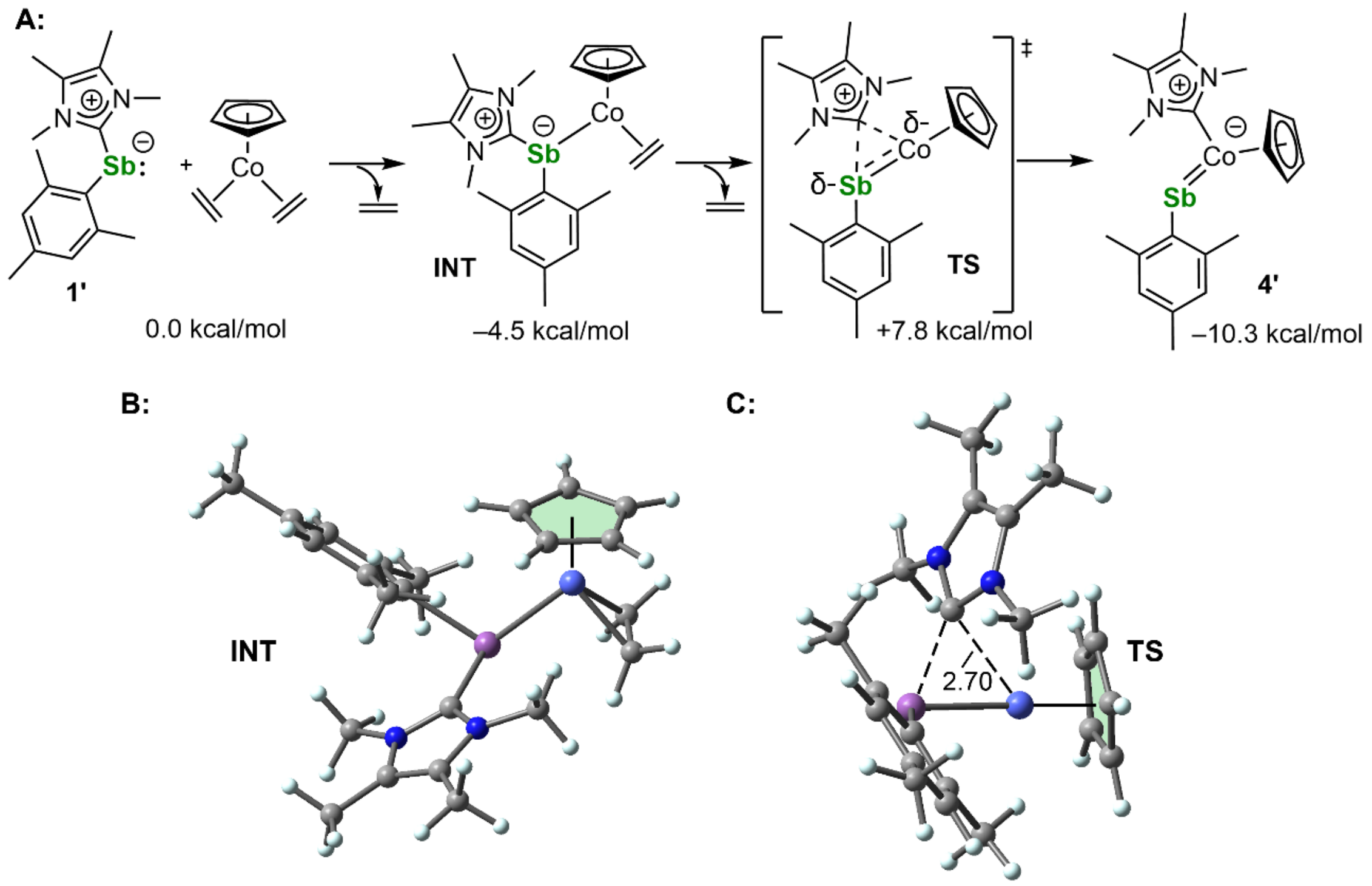

Figure S14. A: Proposed mechanism for the formation of complex **4** from **1**. B: Optimized geometry of the intermediate (INT). C: Optimized geometry of the transition state. Distance in Å.

6. Single-crystal X-ray diffraction analysis

Suitable single crystals of the compounds **2**, **3**, **4**, **5**, **6**, and **7** for X-ray diffraction studies were grown as follows: clear orange plates of **2** and orange blocks of **3** were obtained *via* slow evaporation of their saturated solutions in MeCN/Et_2O (v/v) mixture at –30 °C, brown blocks of **4** were obtained upon cooling of its saturated solution in *n*-pentane at –30 °C, clear brown-orange block shaped crystals of **5** were obtained by slow evaporation of its saturated solution in benzene at room temperature, clear light green block shaped crystals of **6** were obtained by slow evaporation of its saturated solution in *n*-hexane at –30 °C and clear yellow block shaped crystals of **7** were obtained by slow evaporation of its saturated solution in *n*-hexane at –30 °C.

The crystals were protected with Fomblin® Y inside the glove box before mounting on the goniometer.

The data collections of **2**, **4** and **5** were performed on a STOE IPDS-2T 2-circle diffractometer equipped with a low-temperature device (100(2) K, Oxford Cryostream 700er series, Oxford Cryosystems) by using Mo-Kα-radiation (λ = 0.71073 Å, ASTIX++-optics) and an image-plate (34 cm diameter) detector system. Intensities were measured by fine-slicing ω-scans and corrected for background, polarization and Lorentz effects. A semi-empirical absorption correction by scaling of reflection intensities with a subsequent spherical absorption correction was performed with LANA.[24]

Data collection of **3**, **6**, and **7** was performed on BRUKER D8-VENTURE diffractometer using Mo-Kα(λ = 0.71073 Å, mirror optics) using a Photon III CE14 detector. The diffractometer was equipped with low–temperature devices Cryostream 800 series (Oxford Cryosystems), 100 K. Intensities were measured by fine-slicing φ- and ω-scans and corrected for background, polarization and Lorentz effects. Absorption corrections were performed by the empirical method implemented in SADABS 2016/2.[25] OLEX2 was used for data finalization and visualization.[26]

The structures were solved by intrinsic phasing methods and refined by the least-squares procedure implemented in the SHELX program suite. Hydrogen atoms were placed in geometrically calculated positions and included in the refinement process using a riding model on the bound carbon atom.[27, 28]

Special treatments/procedures (side occupancies and masked solvent contributions) are given below.

CCDC numbers CCDC-2547035 (**2**), CCDC-2547036 (**3**), CCDC-2547037 (**4**), CCDC-2547038 (**5**), 2561875 (**6**), and 2561876 (**7**) contain the supplementary crystallographic data for this paper, which can be obtained free of charge from the Cambridge Crystallographic Data Centre via http://www.ccdc.cam.ac.uk/data request/cif.

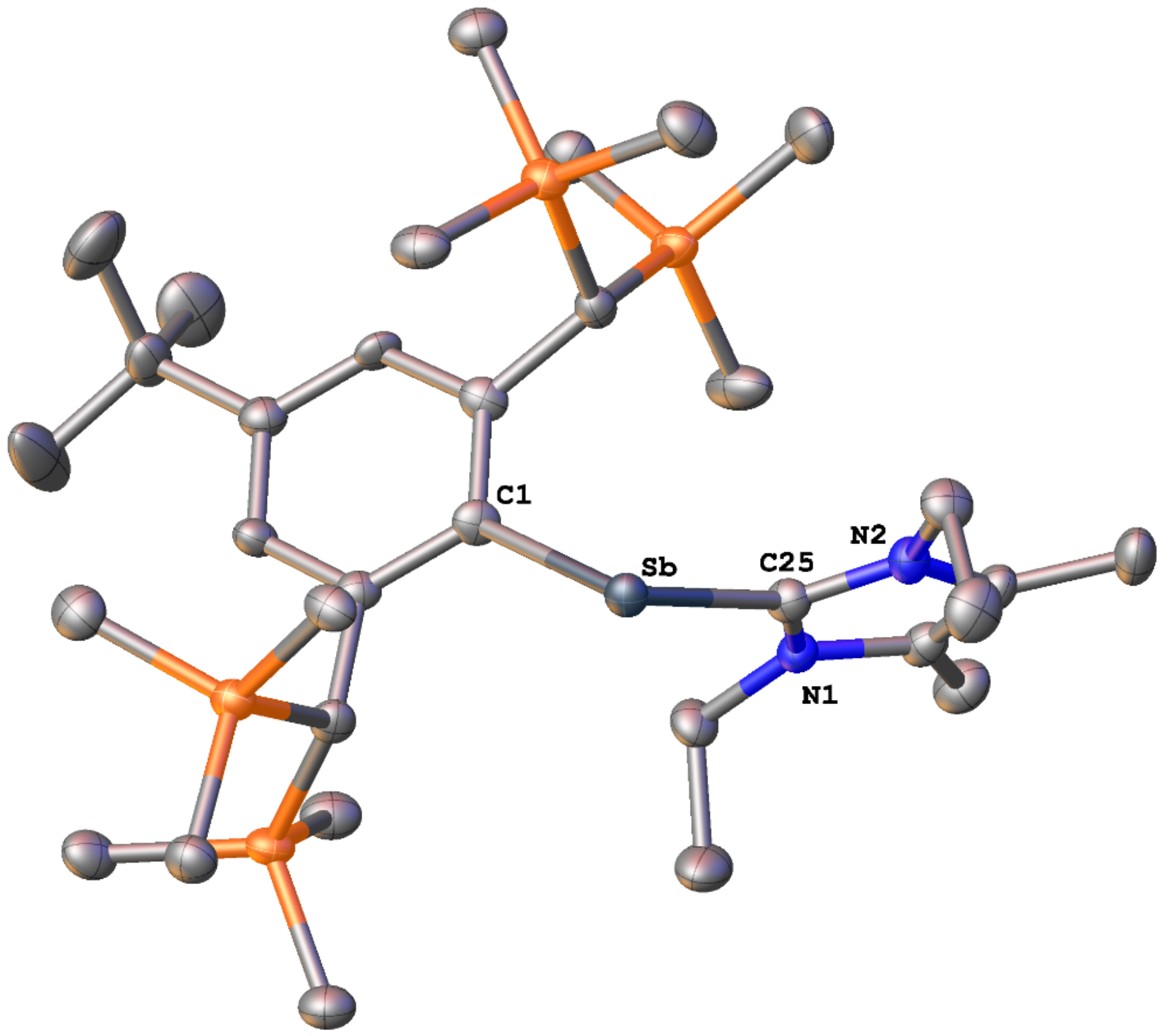

Figure S15. Solid state structure of **2**. Thermal ellipsoids are set to 50% probability level. The second independent molecule in the asymmetric unit is omitted for clarity.

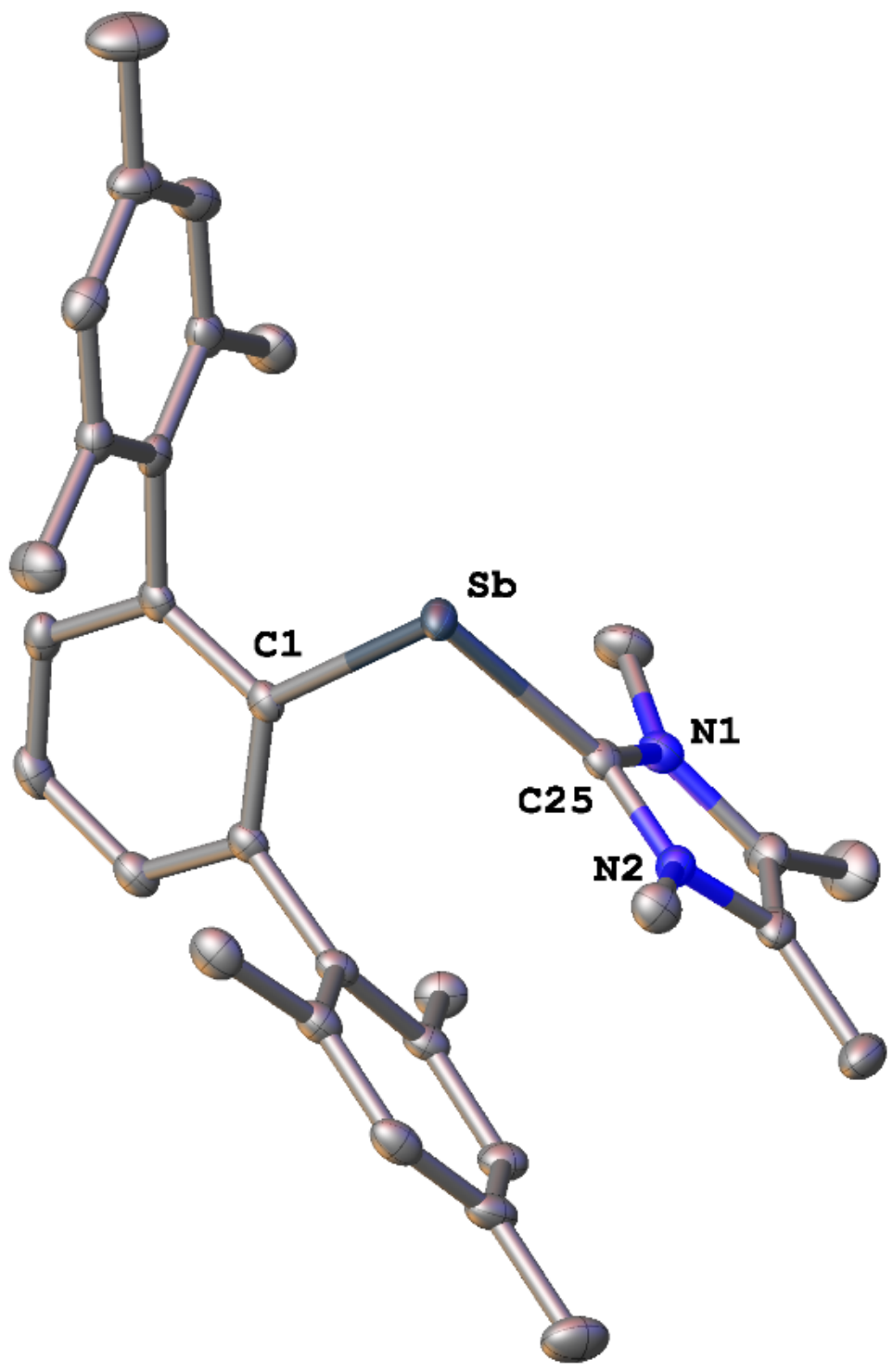

Figure S16. Solid state structure of **3**. Thermal ellipsoids are set to 50% probability level.

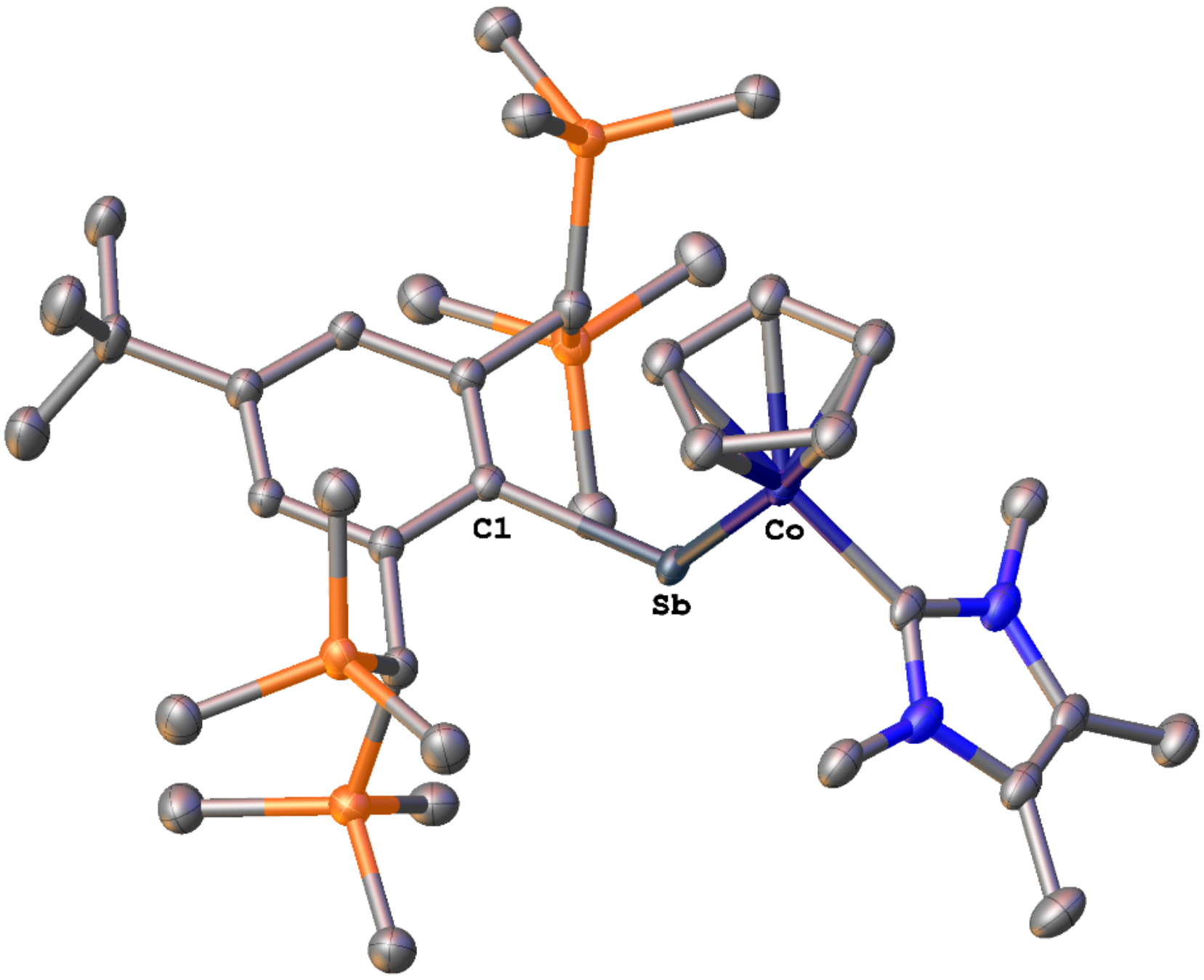

Figure S17. Solid state structure of **4**. Thermal ellipsoids are set to 50% probability level.

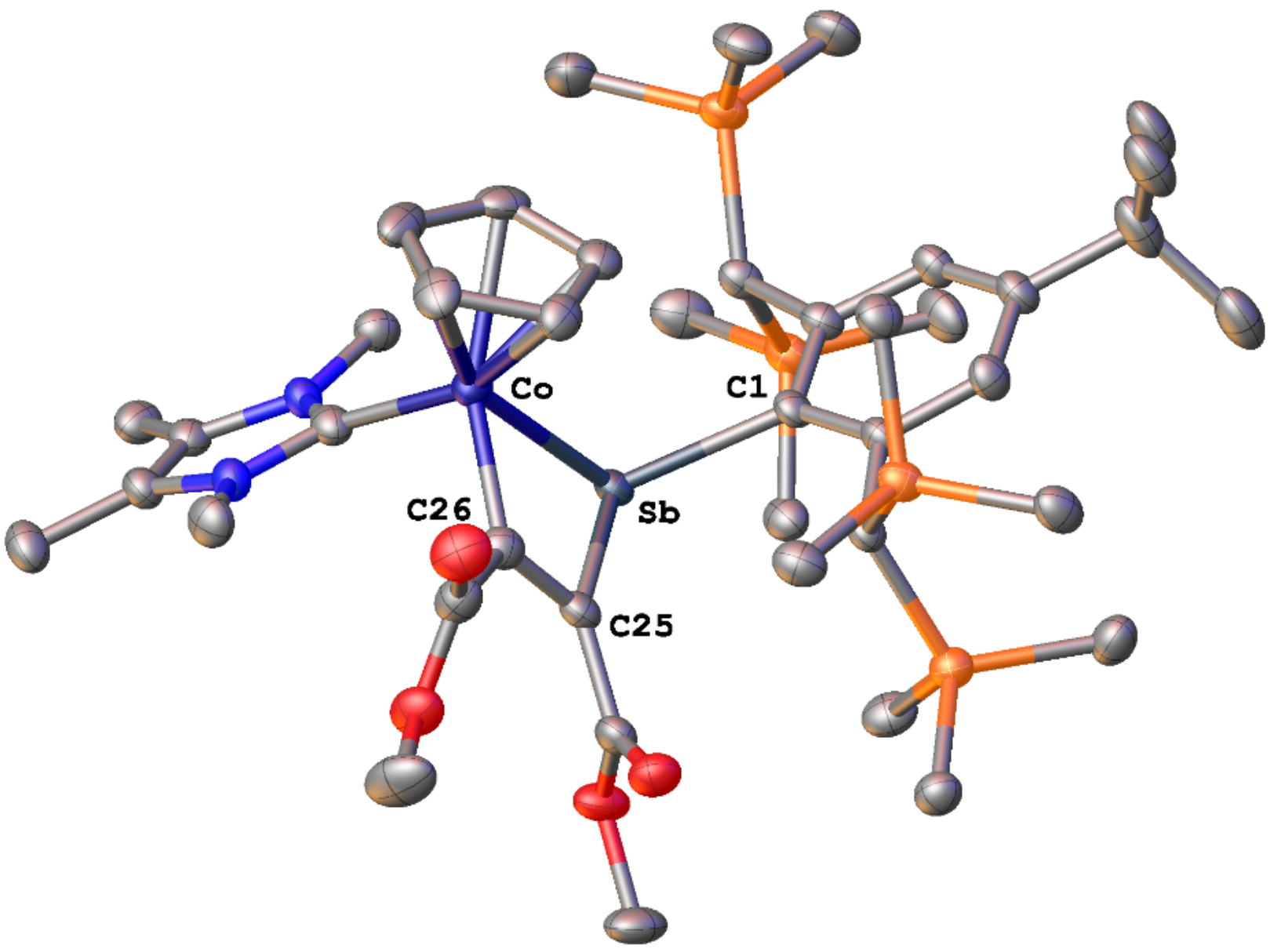

Figure S18. Solid state structure of **5**. Thermal ellipsoids are set to 50% probability level. A co-crystallized benzene molecule and a site occupancy at the *t*-Butyl group (BASF of 17%) is omitted for clarity.

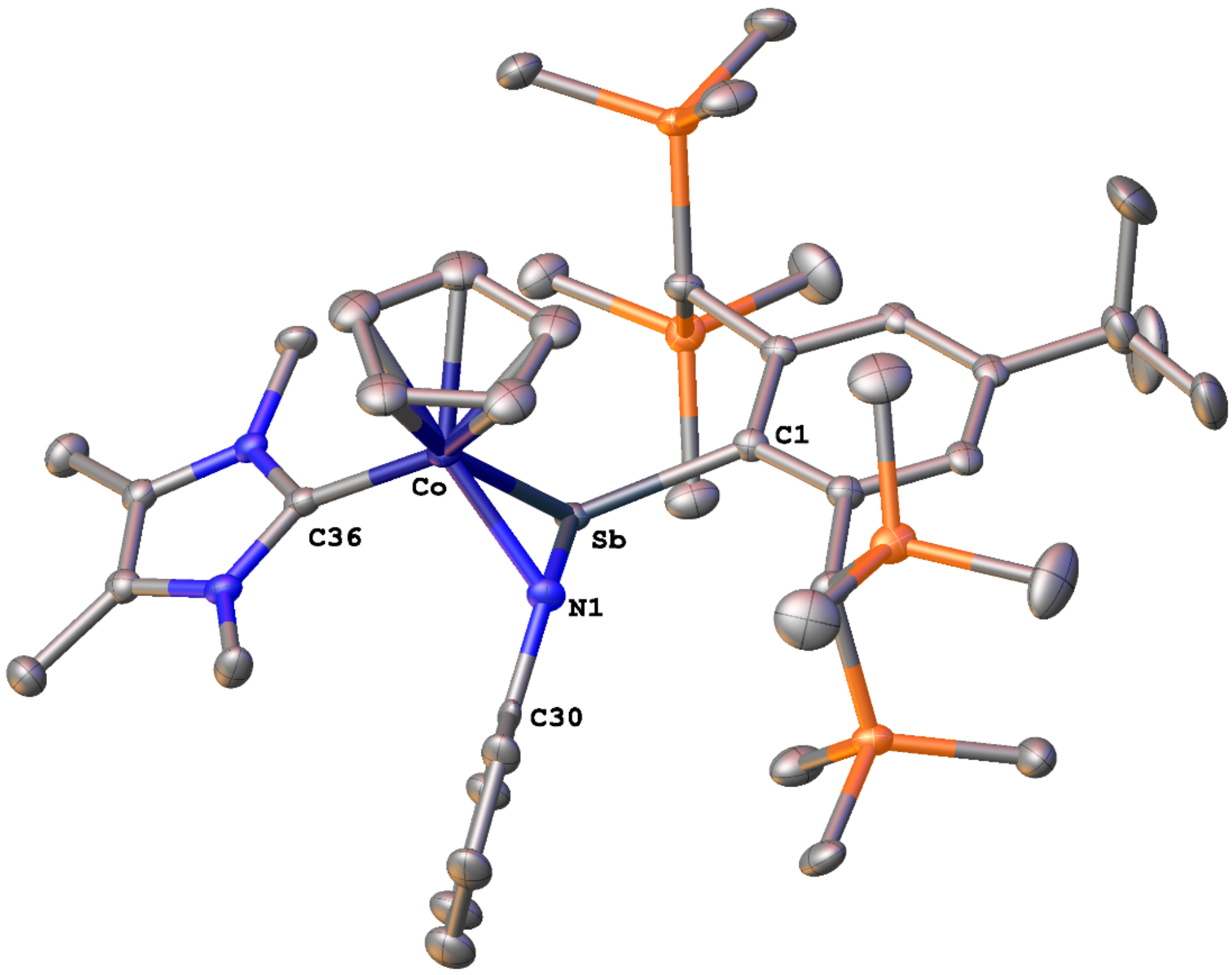

Figure S19. Solid state structure of **6**. Thermal ellipsoids are set to 50% probability level.

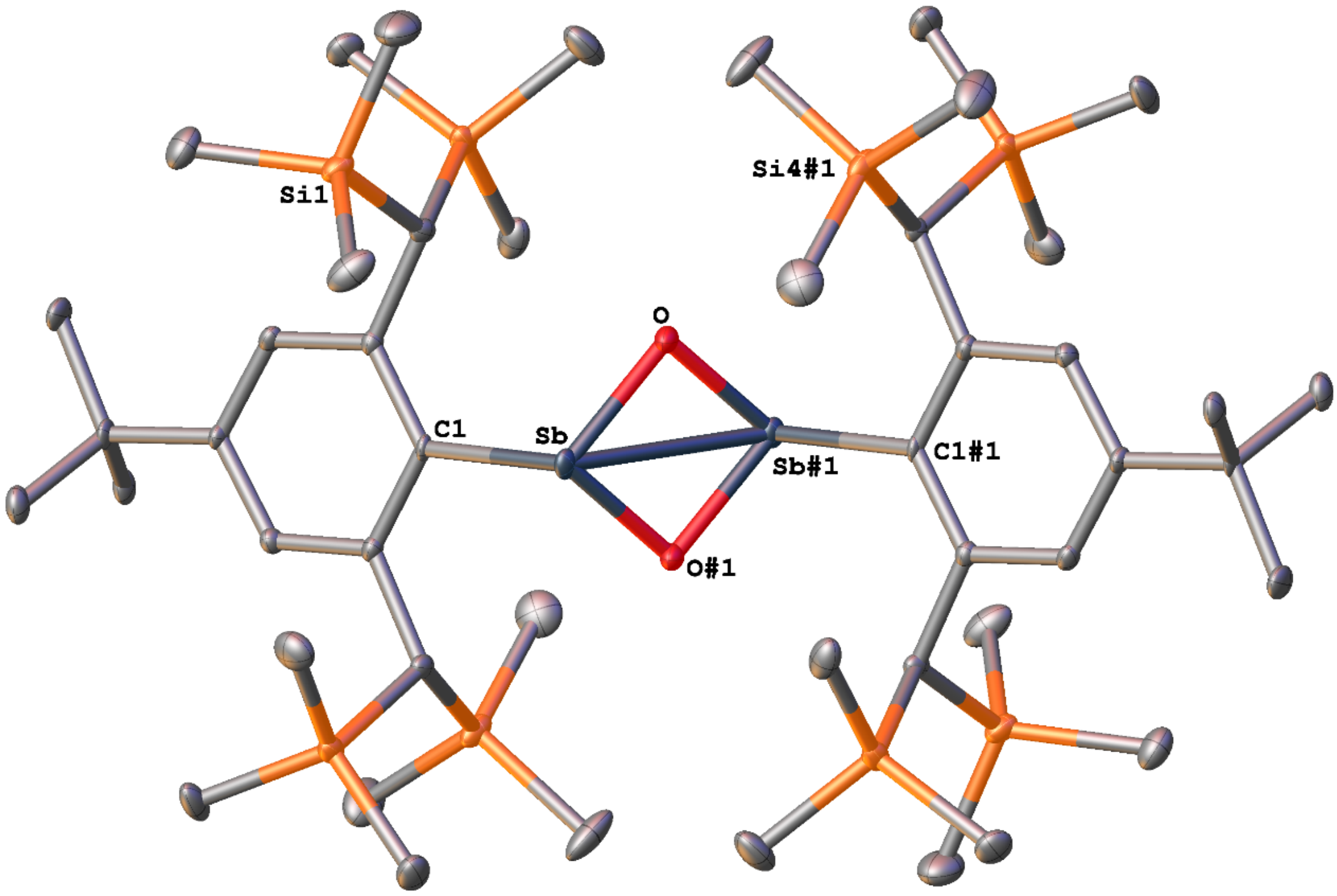

Figure S20. Solid state structure of **7**. Thermal ellipsoids are set to 50% probability level.

7. NMR spectra

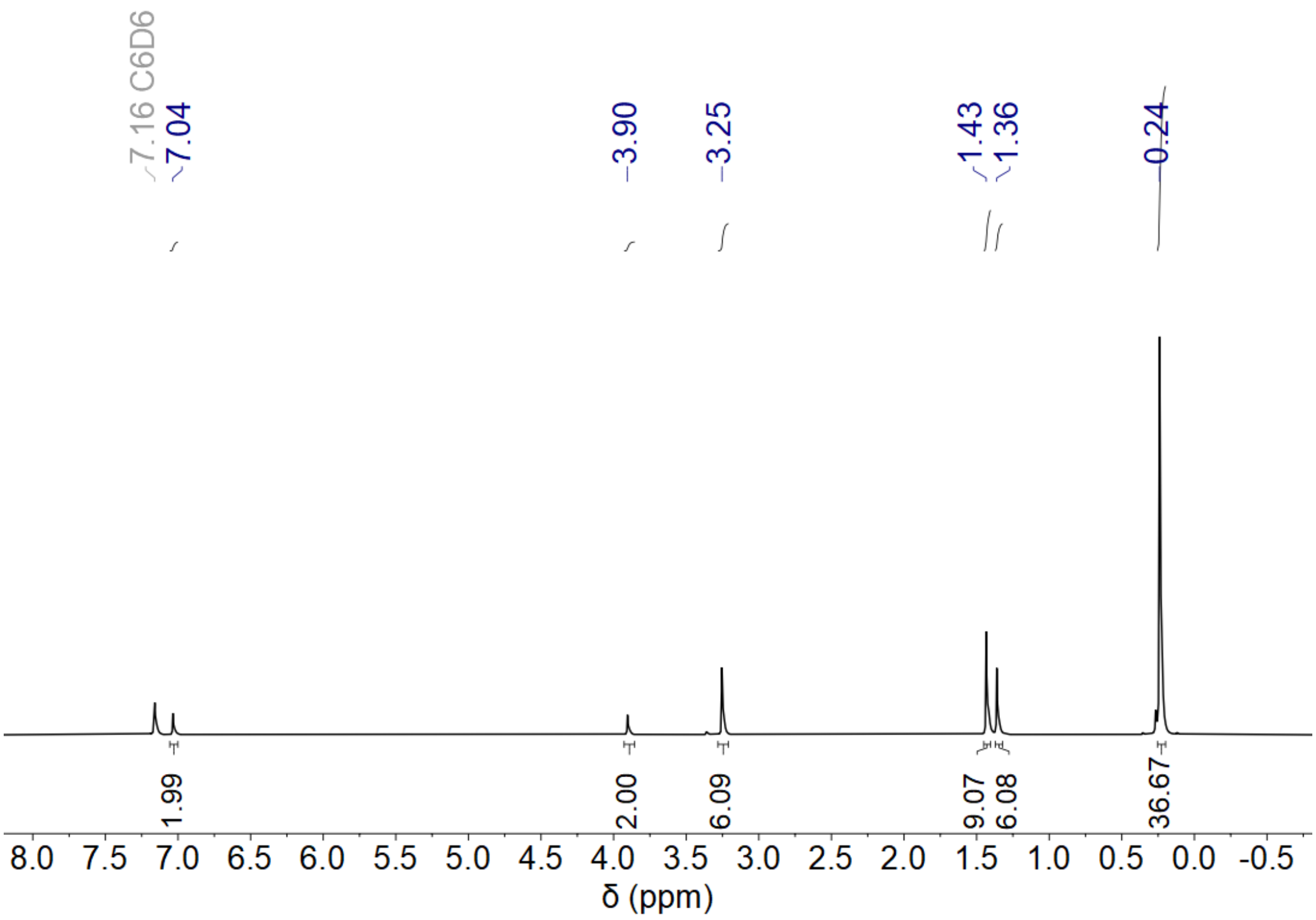

¹H NMR (500 MHz) spectrum of complex 1 in C_6D_6.

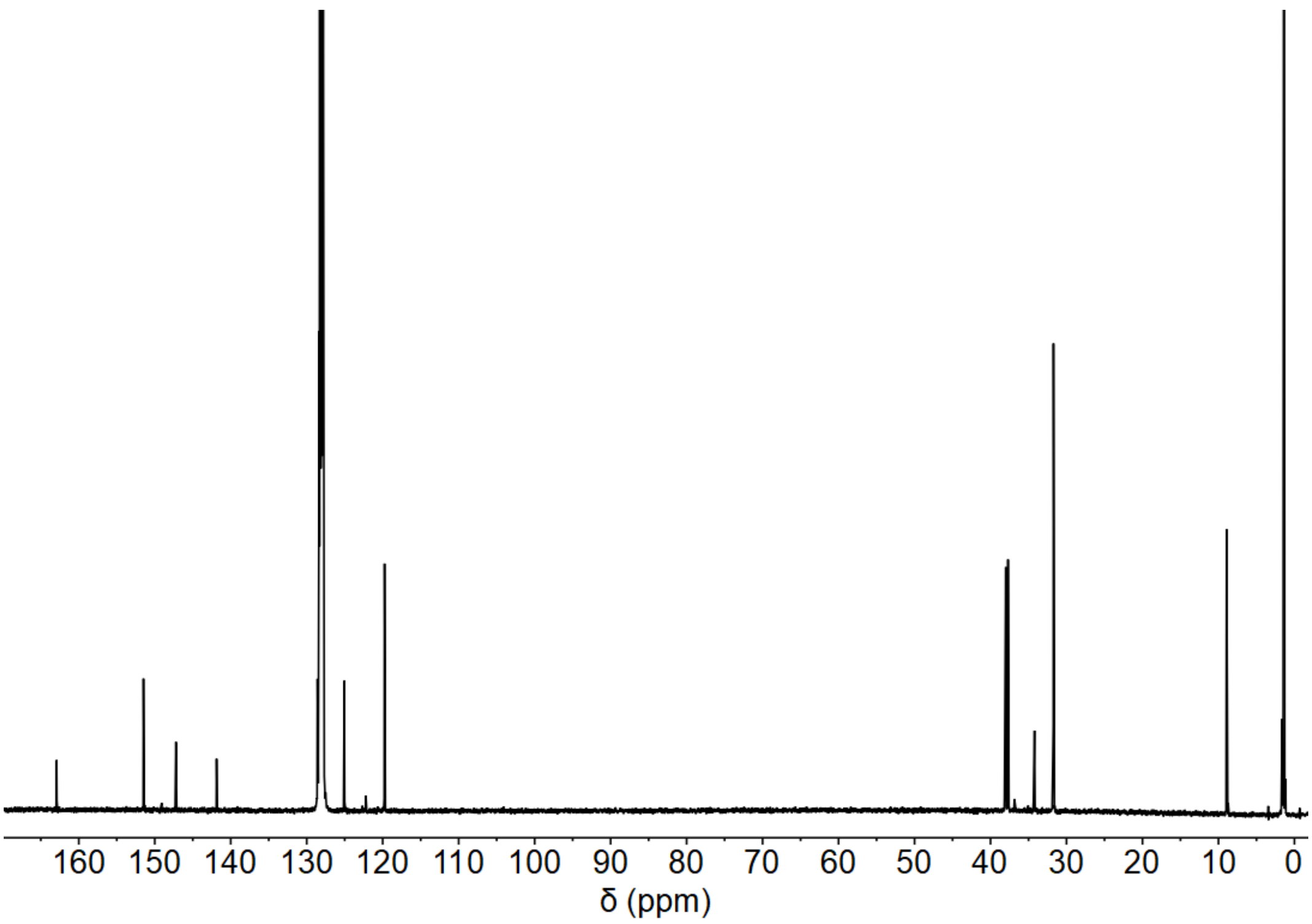

¹³C{¹H} NMR (126 MHz) spectrum of complex 1 in C_6D_6.

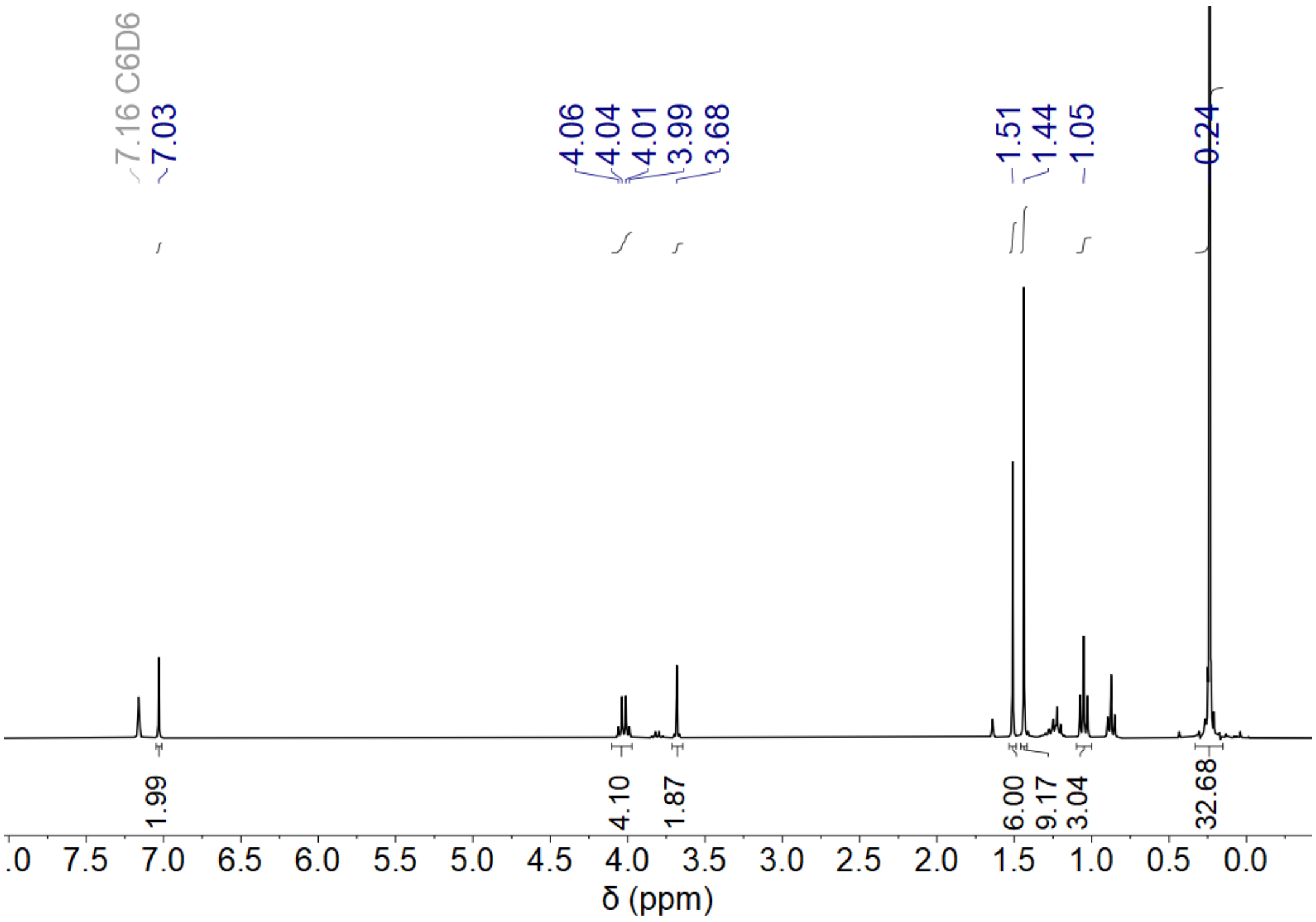

^{1}H NMR (500 MHz) spectrum of complex 2 in C_6D_6.

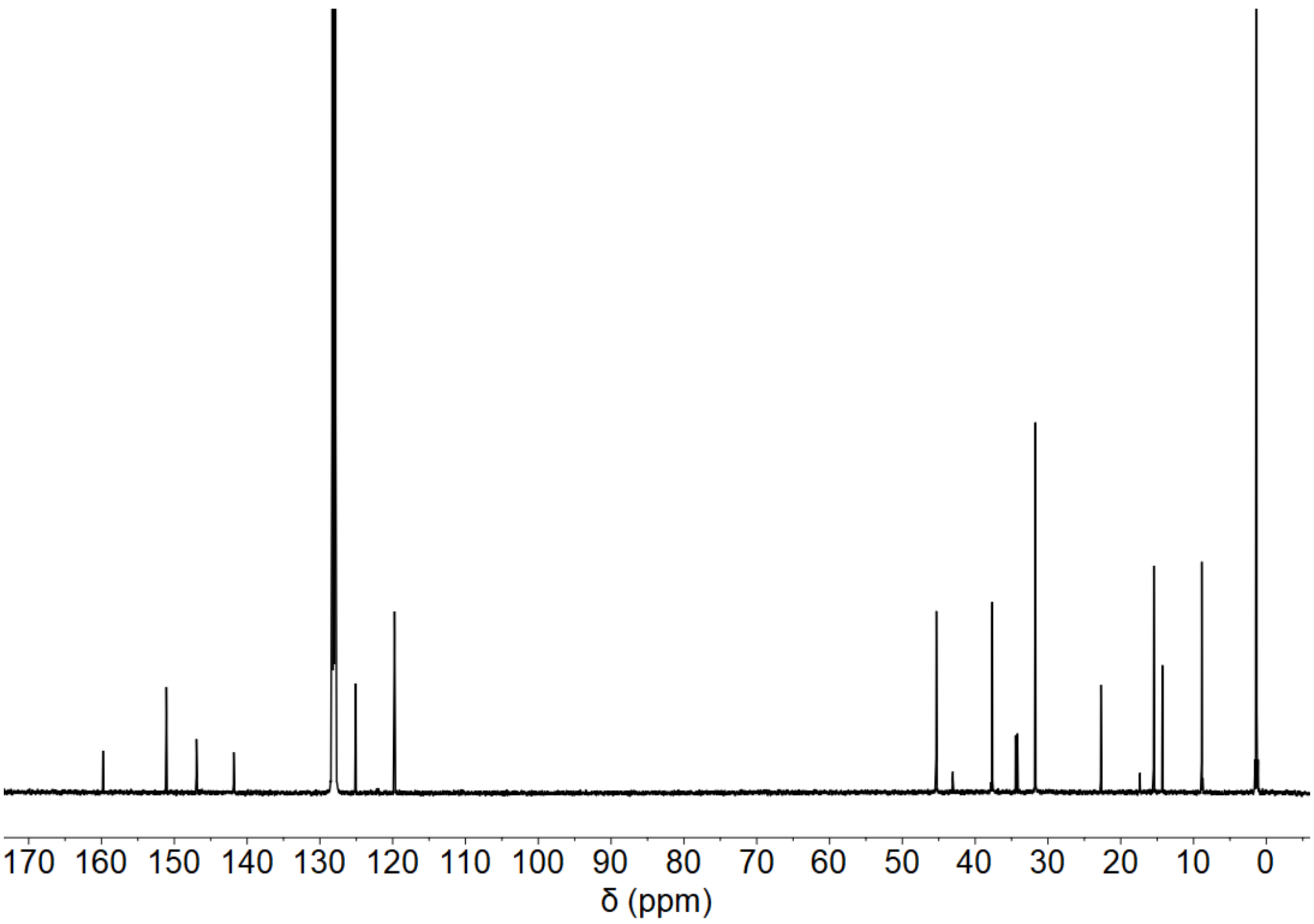

$^{13}C\{^1H\}$ NMR (126 MHz) spectrum of complex 2 in C_6D_6.

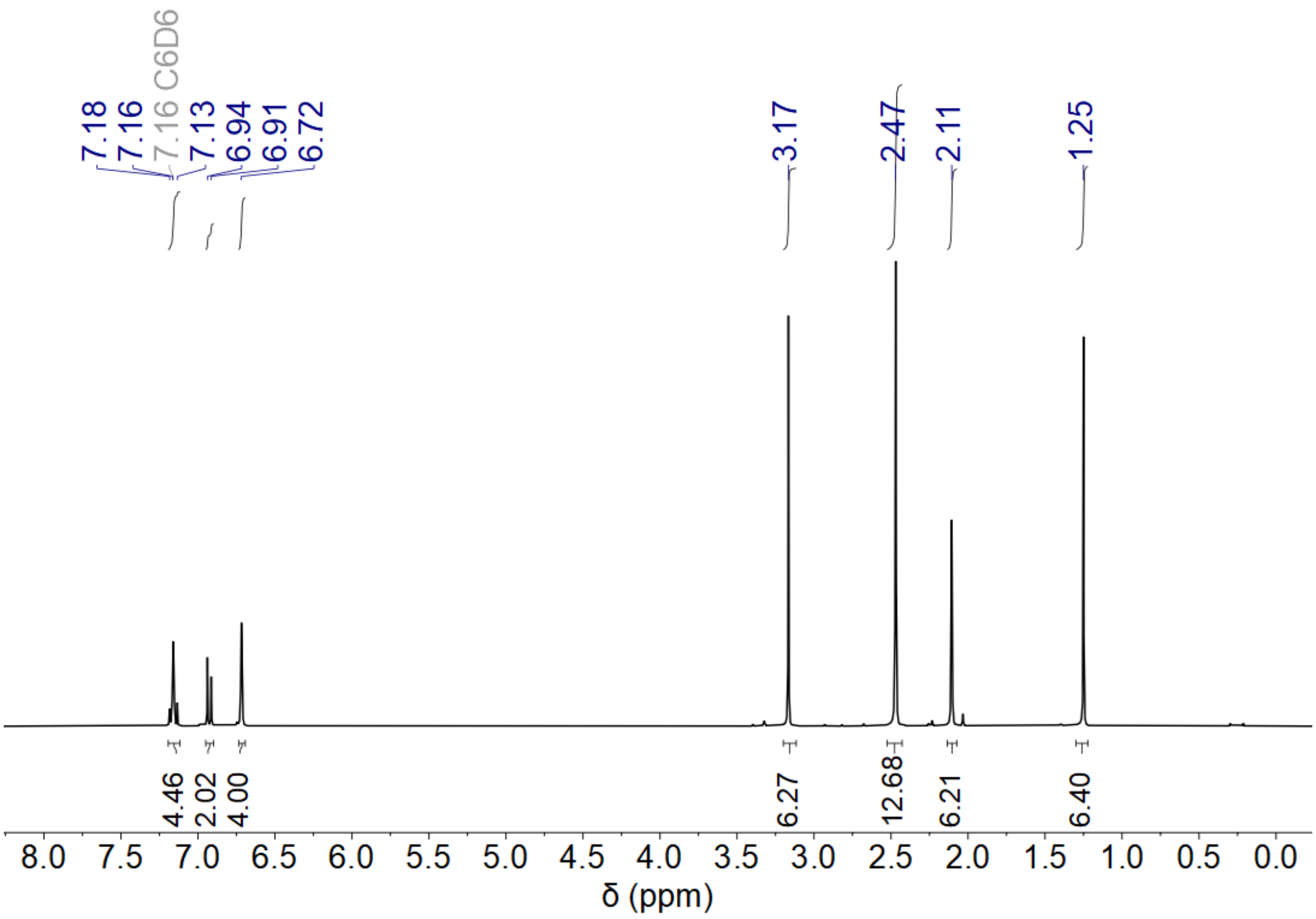

^{1}H NMR (500 MHz) spectrum of complex 3 in C_6D_6.

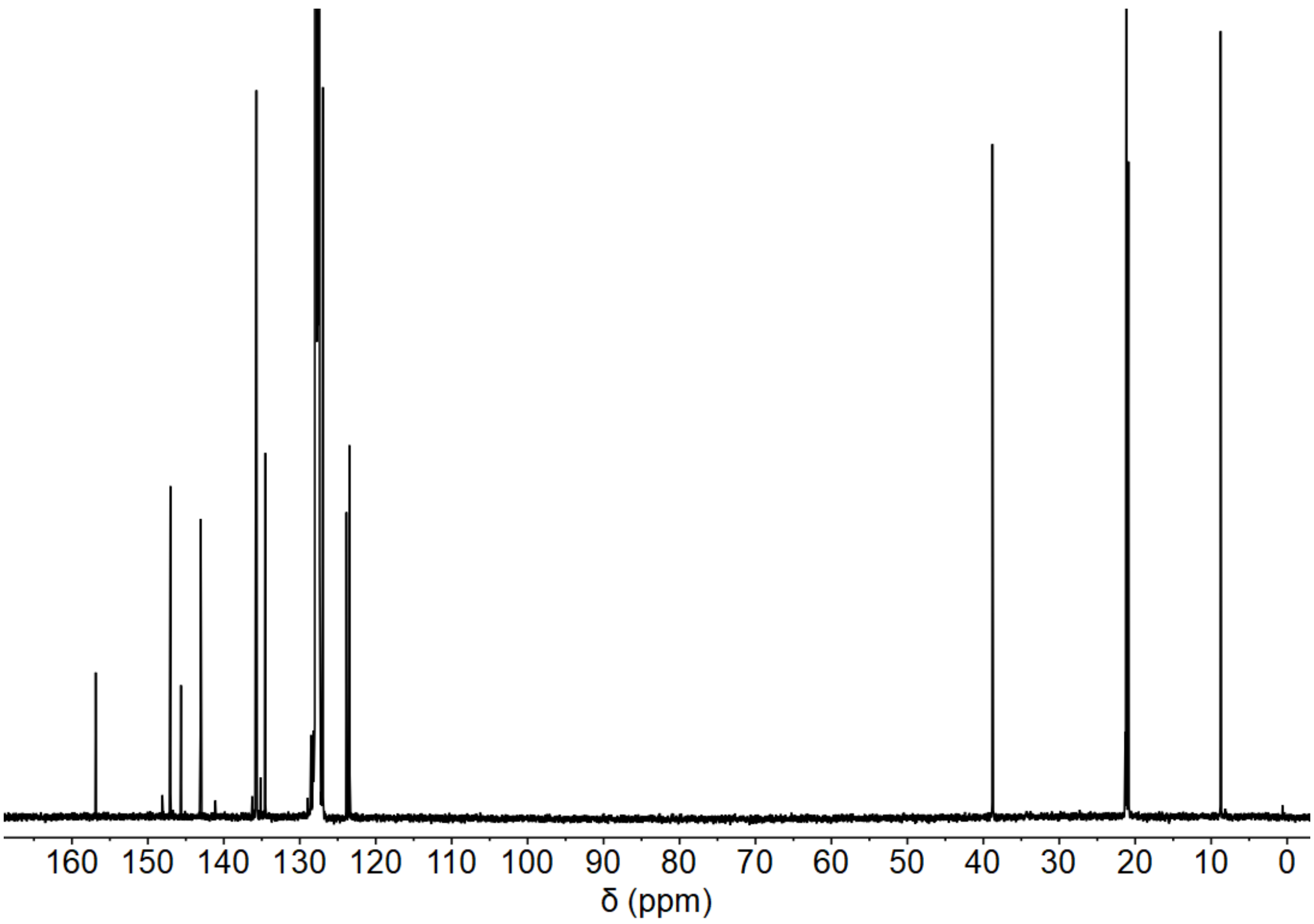

^{13}C{^{1}H} NMR (126 MHz) spectrum of complex 3 in C_6D_6.

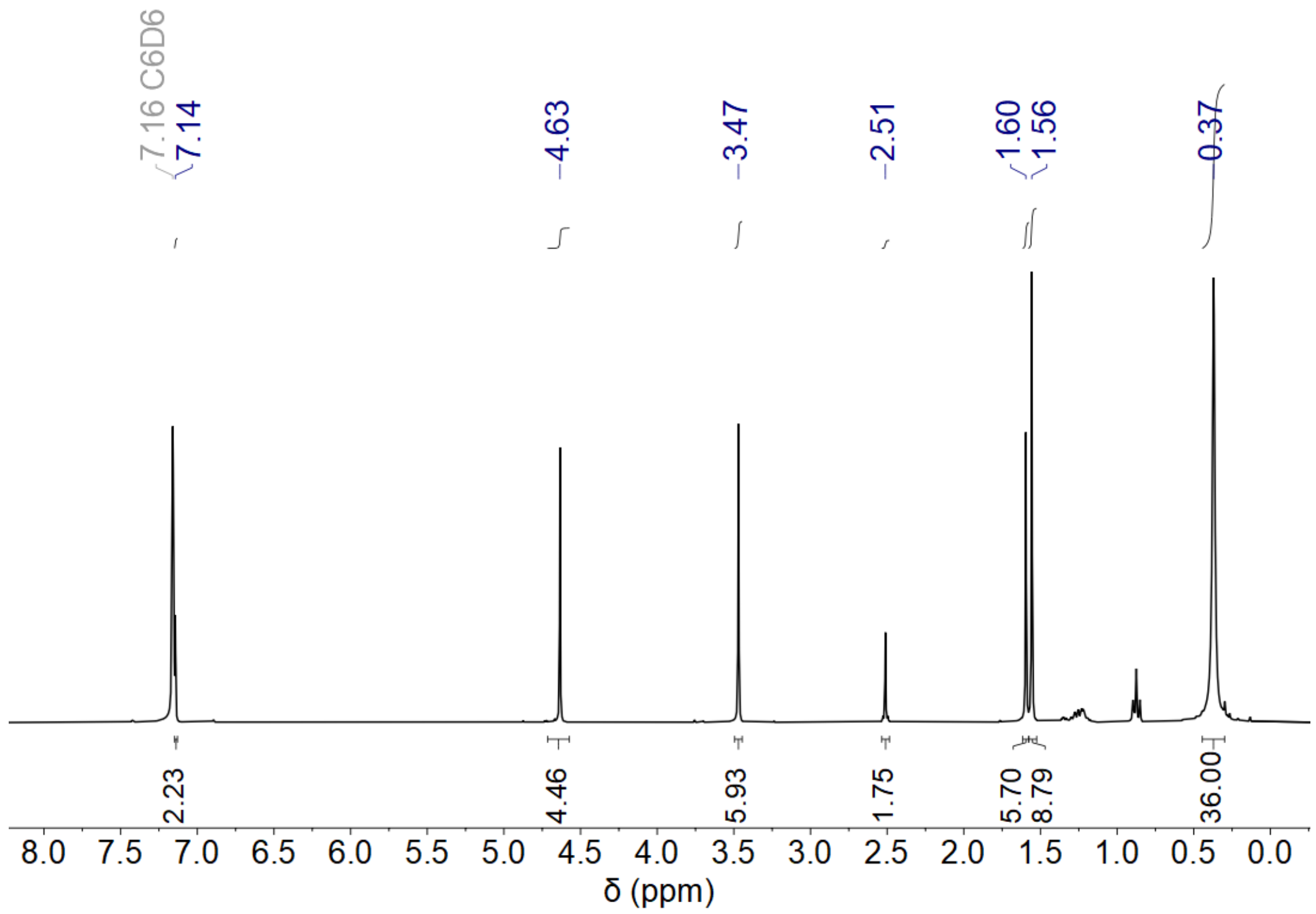

^{1}H NMR (500 MHz) spectrum of complex 4 in C_6D_6.

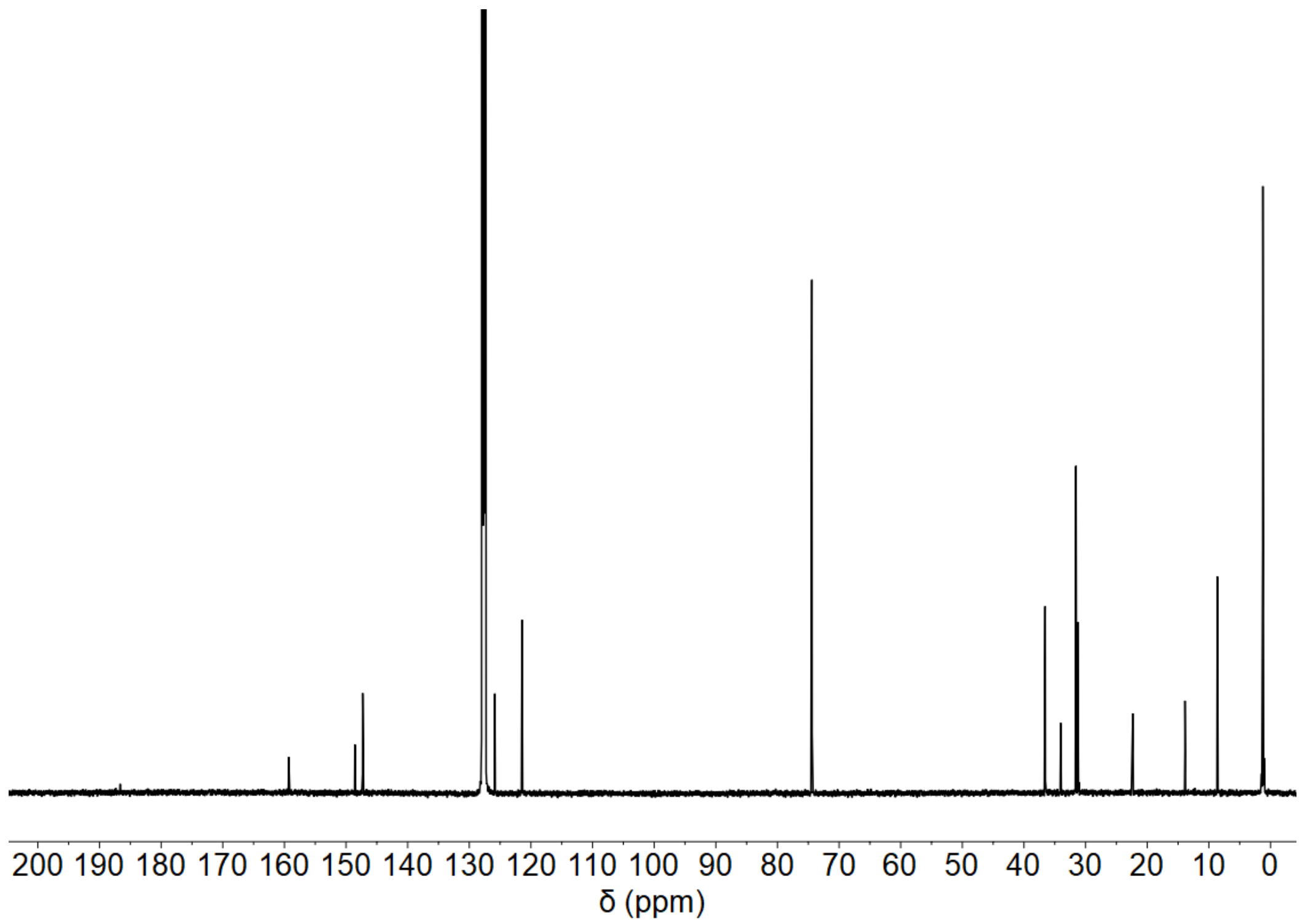

$^{13}C\{^{1}H\}$ NMR (126 MHz) spectrum of complex 4 in C_6D_6.

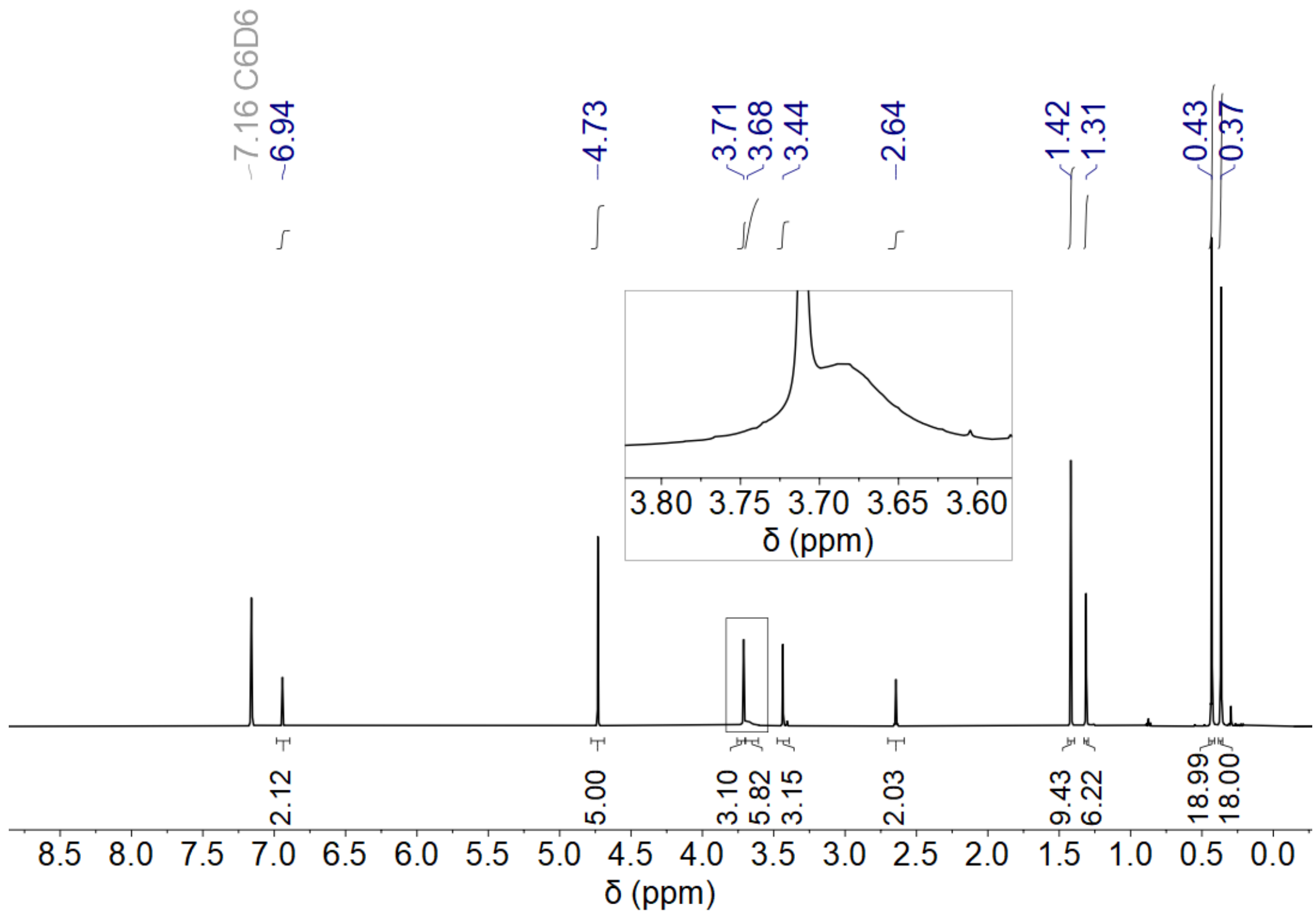

^{1}H NMR (500 MHz) spectrum of complex 5 in C_6D_6.

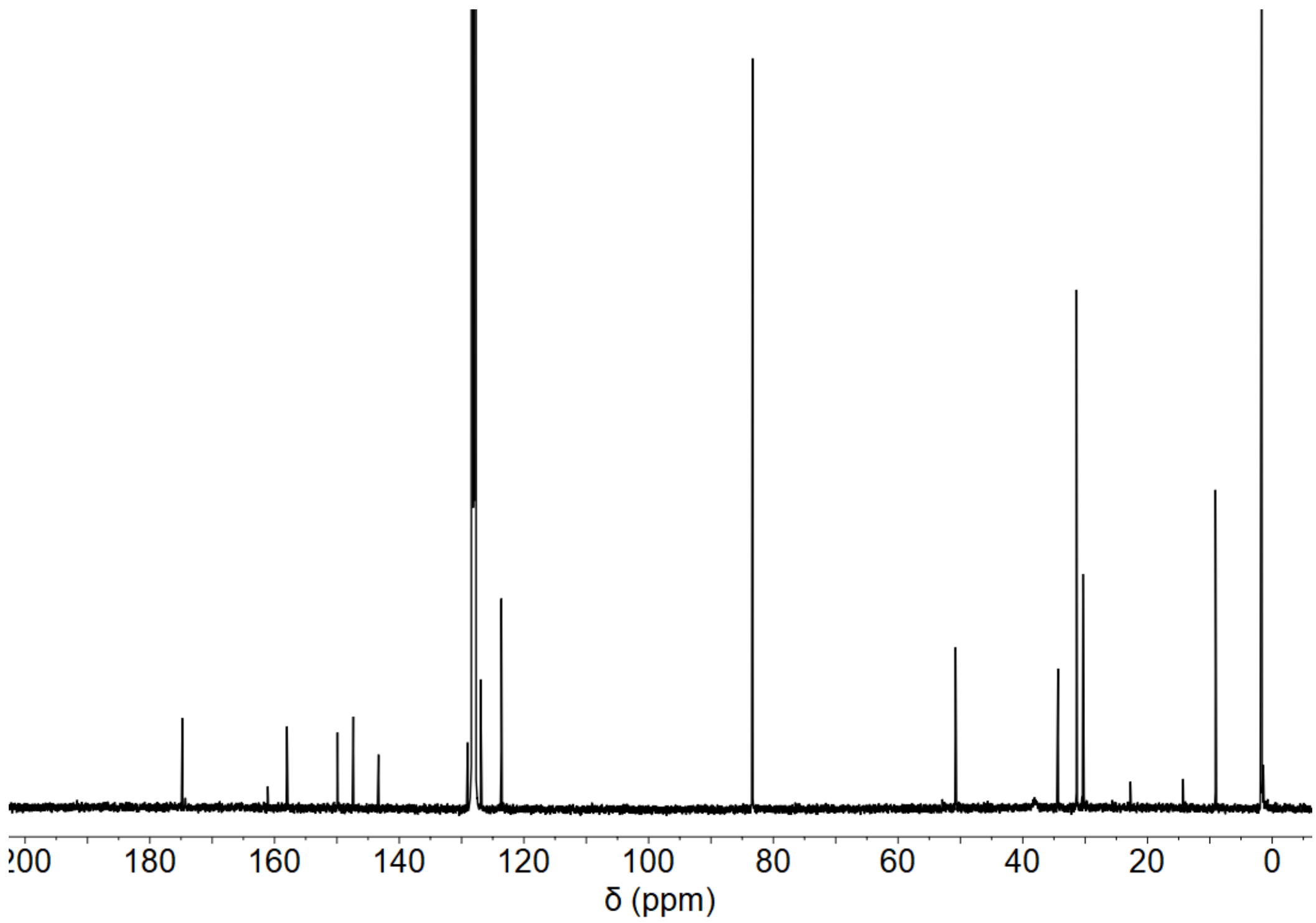

$^{13}C\{^{1}H\}$ NMR (126 MHz) spectrum of complex 5 in C_6D_6.

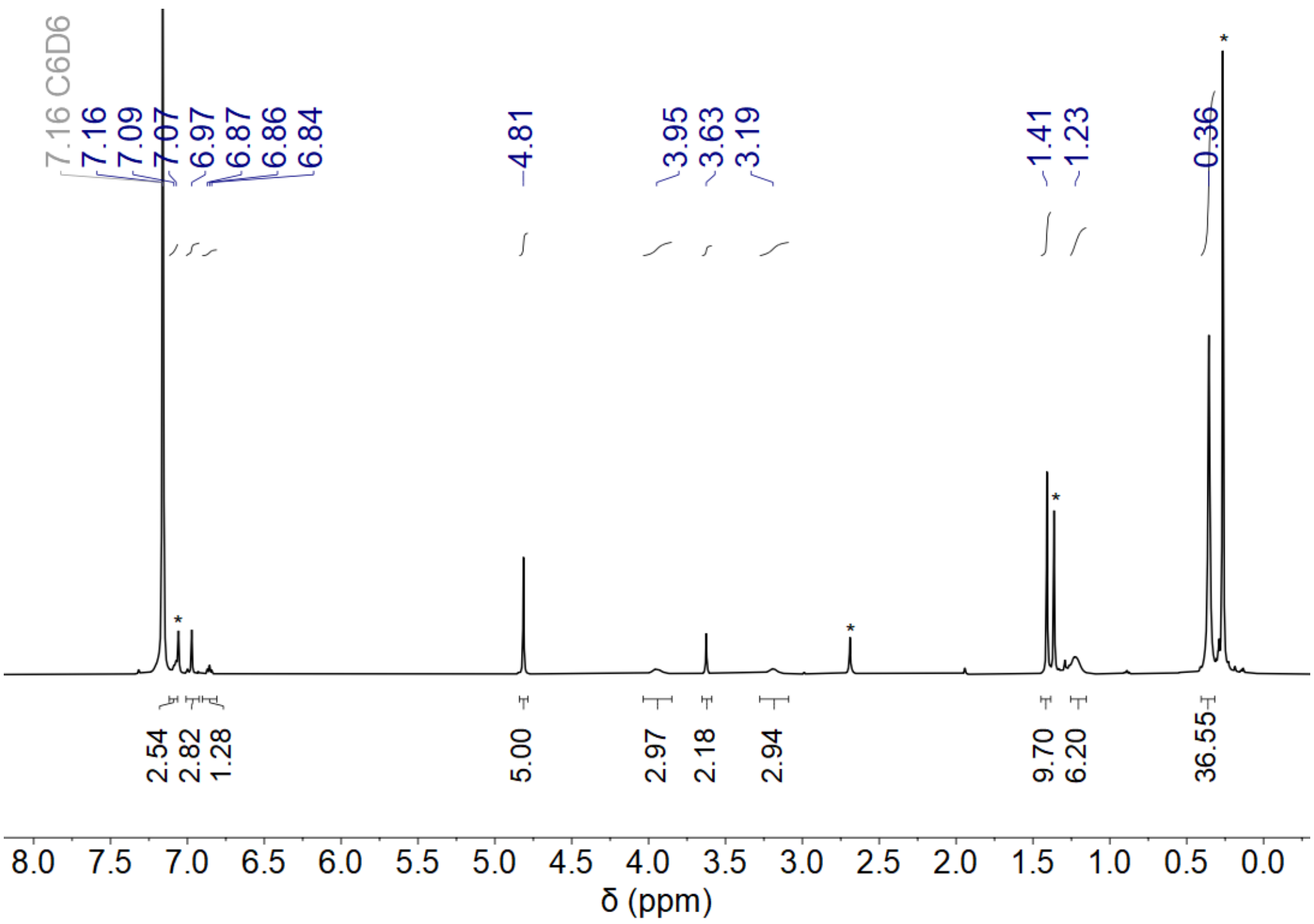

^{1}H NMR (500 MHz) spectrum of complex 6 in C_6D_6. Signals marked with * correspond to complex 7.

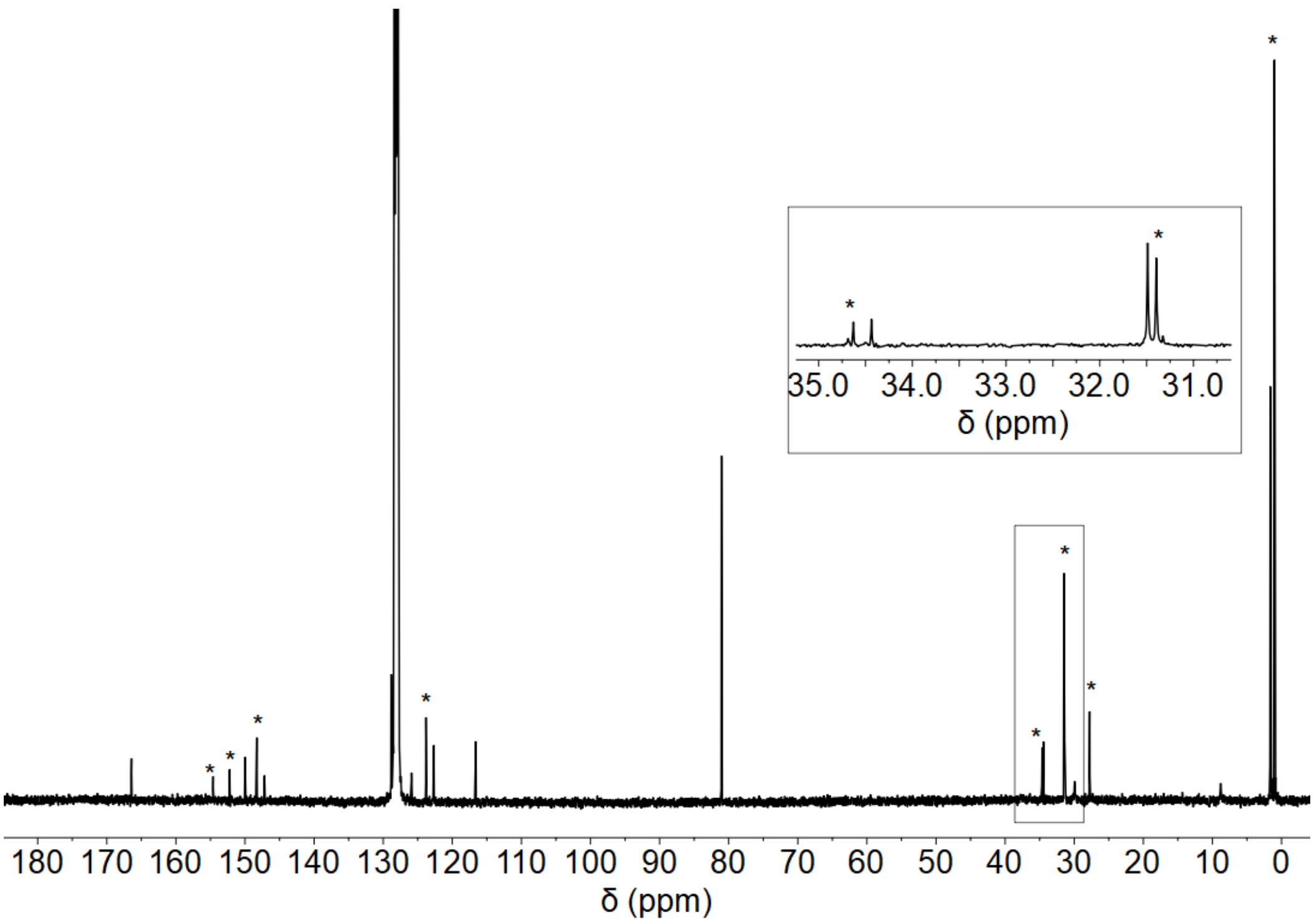

^{13}C{^{1}H} NMR (126 MHz) spectrum of complex 6 in C_6D_6. Signals marked with * correspond to complex 7.

8. References

[1] D. Meleschko, P. Palui, R. M. Gomila, G. Schnakenburg, A. C. Filippou, A. Frontera, A. Bismuto, Light-Dependent Reactivity of Heavy Pnictogen Double Bonds, *Angewandte Chemie International Edition* **2024**, *63*, e202405400.

[2] B. Twamley, C. D. Sofield, M. M. Olmstead, P. P. Power, Homologous Series of Heavier Element Dipnictenes 2,6-$Ar_2H_3C_6E{=}EC_6H_3$-2,6-Ar_2 (E = P, As, Sb, Bi; Ar = Mes = C_6H_2-2,4,6-Me_3; or Trip = C_6H_2-2,4,6-iPr_3) Stabilized by m-Terphenyl Ligands, *Journal of the American Chemical Society* **1999**, *121*, 3357-3367.

[3] N. Kuhn, T. Kratz, Synthesis of Imidazol-2-ylidenes by Reduction of Imidazole-2(3H)-thiones, *Synthesis* **1993**, *1993*, 561-562.

[4] K. Jonas, E. Deffense, D. Habermann, Synthesis and Reactions of η^5-Cyclopentadienylbis(ethene)cobalt, *Angewandte Chemie International Edition in English* **1983**, *22*, 716-717.

[5] J. Tao, J. P. Perdew, V. N. Staroverov, G. E. Scuseria, Climbing the Density Functional Ladder: Nonempirical Meta--Generalized Gradient Approximation Designed for Molecules and Solids, *Physical Review Letters* **2003**, *91*, 146401.

[6] S. Grimme, S. Ehrlich, L. Goerigk, Effect of the damping function in dispersion corrected density functional theory, *Journal of computational chemistry* **2011**, *32*, 1456-1465.

[7] F. Weigend, R. Ahlrichs, Balanced basis sets of split valence, triple zeta valence and quadruple zeta valence quality for H to Rn: Design and assessment of accuracy, *Physical Chemistry Chemical Physics* **2005**, *7*, 3297-3305.

[8] F. Weigend, Accurate Coulomb-fitting basis sets for H to Rn, *Physical Chemistry Chemical Physics* **2006**, *8*, 1057-1065.

[9] F. Furche, R. Ahlrichs, C. Hättig, W. Klopper, M. Sierka, F. Weigend, Turbomole, *WIREs Computational Molecular Science* **2014**, *4*, 91-100.

[10] P. Palui, S. Ghosh, R. M. Gomila, G. Schnakenburg, A. Frontera, A. Bismuto, Combining Distibene, Diazoolefins, and Visible Light: Synthesis and Reactivity of Inorganic Rings, *Journal of the American Chemical Society* **2025**, *147*, 1421-1426.

[11] D. Meleschko, P. Palui, G. Pugliese, G. Schnakenburg, R. M. Gomila, A. Frontera, A. Bismuto, Synthesis, Reactivity, and Theoretical Insights of Co-Sb_2 and Co-Bi_2 Rings, *Organometallics* **2024**, *43*, 2581-2588.

[12] F. Neese, Software update: The ORCA program system—Version 5.0, *WIREs Computational Molecular Science* **2022**, *12*, e1606.

[13] D. Peng, N. Middendorf, F. Weigend, M. Reiher, An efficient implementation of two-component relativistic exact-decoupling methods for large molecules, *The Journal of Chemical Physics* **2013**, *138*, 184105.

[14] Y. J. Franzke, R. Treß, T. M. Pazdera, F. Weigend, Error-consistent segmented contracted all-electron relativistic basis sets of double- and triple-zeta quality for NMR shielding constants, *Physical Chemistry Chemical Physics* **2019**, *21*, 16658-16664.

[15] S. Kossmann, F. Neese, Efficient Structure Optimization with Second-Order Many-Body Perturbation Theory: The RIJCOSX-MP2 Method, *Journal of Chemical Theory and Computation* **2010**, *6*, 2325-2338.

[16] G. L. Stoychev, A. A. Auer, F. Neese, Automatic Generation of Auxiliary Basis Sets, *Journal of Chemical Theory and Computation* **2017**, *13*, 554-562.

[17] G. Knizia, Intrinsic Atomic Orbitals: An Unbiased Bridge between Quantum Theory and Chemical Concepts, *Journal of Chemical Theory and Computation* **2013**, *9*, 4834-4843.

[18] E. D. Glendening, C. R. Landis, F. Weinhold, NBO 7.0: New vistas in localized and delocalized chemical bonding theory, *Journal of computational chemistry* **2019**, *40*, 2234-2241.

[19] I. Mayer, Bond order and valence indices: A personal account, *Journal of computational chemistry* **2007**, *28*, 204-221.

[20] K. B. Wiberg, Application of the pople-santry-segal CNDO method to the cyclopropylcarbinyl and cyclobutyl cation and to bicyclobutane, *Tetrahedron* **1968**, *24*, 1083-1096.

[21] M. P. Mitoraj, A. Michalak, T. Ziegler, A Combined Charge and Energy Decomposition Scheme for Bond Analysis, *Journal of Chemical Theory and Computation* **2009**, *5*, 962-975.

[22] T. Lu, F. Chen, Multiwfn: A multifunctional wavefunction analyzer, *Journal of computational chemistry* **2012**, *33*, 580-592.

[23] T. K. R. Dennington, J. Millam, GaussView Version 6.1.1 ed., Semichem Inc., Shawnee Mission, KS, **2019**.

[24] J. Koziskova, F. Hahn, J. Richter, J. Kožíšek, Comparison of different absorption corrections on the model structure of tetrakis(μ_2-acetato)-diaqua-di-copper(II), *Acta Chimica Slovaca* **2016**, *9*, 136-140.

[25] SADABS-2016/2, Bruker AXS, **2016**.

[26] O. V. Dolomanov, L. J. Bourhis, R. J. Gildea, J. A. K. Howard, H. Puschmann, OLEX2: a complete structure solution, refinement and analysis program, *Journal of Applied Crystallography* **2009**, *42*, 339-341.

[27] G. Sheldrick, Crystal structure refinement with SHELXL, *Acta Crystallographica Section C* **2015**, *71*, 3-8.

[28] G. Sheldrick, SHELXT - Integrated space-group and crystal-structure determination, *Acta Crystallographica Section A* **2015**, *71*, 3-8.
